# Computational Electrosynthesis: A Perspective on Mechanistic Questions, Methodological Approaches, and Elucidating the Role of the Electrical Double Layer

**DOI:** 10.1021/acs.jpcc.5c04993

**Published:** 2025-09-18

**Authors:** John H. Hymel, Chloe A. Renfro, Shahriar N. Khan, John P. Pederson, Jesse G. McDaniel

**Affiliations:** School of Chemistry and Biochemistry, 1372 Georgia Institute of Technology, Atlanta, Georgia 30332-0400, United States

## Abstract

In this perspective, we discuss how modern computational chemistry techniques are utilized to elucidate electrosynthesis reaction mechanisms via the computation of kinetic rate parameters that depend on reaction conditions. The specific focus is on elementary reaction steps that occur within the electrical double layer (EDL) environment at the working electrode/electrolyte interface, for which the governing free energy profiles and rate constants can be explicitly computed from classical molecular dynamics and hybrid quantum mechanics/molecular mechanics (QM/MM) computer simulations with atomistic resolution of the EDL environment. We focus on direct electrolysis reactions at inert electrodes with prototypical “E-C-E” mechanisms, in which the reaction yield and selectivity may depend on whether the full process occurs heterogeneously within the EDL, or rather the steps following the initial electron transfer proceed homogeneously with the second electron transfer occurring via disproportionation (DISP pathway). For short-lived radical intermediates with lifetimes of ∼10^–10^–10^–7^ s, branching between heterogeneous E-C-E and homogeneous DISP pathways is dictated by rate constants for substrate/intermediate desorption from the electrode surface (e.g., *k*_D_ ∼ 10^7^–10^10^ s^–1^), which are difficult to determine experimentally but can be computed from molecular dynamics simulations. We discuss how strong solvophobic forces within electrolytes lead to significant association free energies between electrogenerated radical ion intermediates and the working electrode surface, resulting in residence times within the EDL that are on par with time constants of fast radical ion reactions. For electrosynthesis reactions that proceed entirely heterogeneously, the EDL environment can substantially modulate the reaction kinetics via electrostatic, solvophobic, and steric forces. We present several case studies highlighting modulated reaction kinetics within the EDL for nucleophilic coupling and deprotonation reactions of cation radical intermediates in anodic electrosynthesis. We expect that computational techniques will play an increasingly valuable role in optimizing electrosynthesis reactions via mechanistic descriptions of how key kinetic rate parameters are modulated by electrochemical reaction conditions.

## Graphical abstract

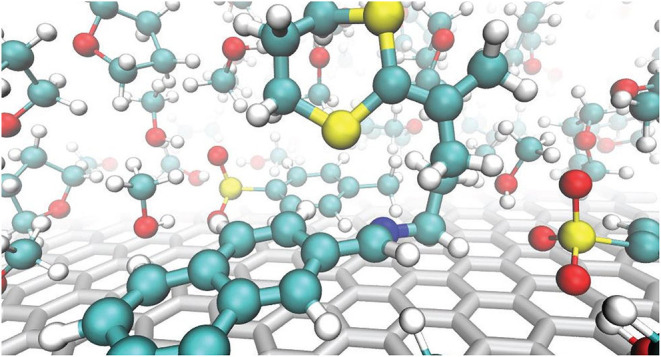

## 1 Introduction

The field of electrosynthesis, or synthetic organic electrochemistry, has historically encompassed primarily organic chemists and electrochemists; in this perspective article, we discuss how modern computational chemistry techniques can contribute to this field through computations of key kinetic rate parameters that depend on the electrochemical reaction conditions. There is substantial, revitalized interest in electrosynthesis methods among the organic chemistry community due to its wide-ranging versatility and access to unique reaction pathways.^1–11^ In this regard, it is broadly the case that developing a better understanding of the electrochemical reaction mechanisms and associated kinetic rate parameters can often lead directly to practical improvements in the reaction yield/selectivity. Many empirical examples have been reported in which tuning the reaction conditions (e.g., solvent, electrolyte, overpotential) lead to dramatic changes in reaction outcome, presumably from altered kinetics of elementary reaction steps (a prototypical example is the electrochemical adiponitrile process^12,13^ ).^1,14–16^ Costentin and Savéant have summarized that, in electrosynthesis, “deciphering the mechanism of the global electrochemical process is thus intimately related to measuring and planning product selectivity”.^17^ This perspective highlights the use of modern computer simulation techniques for predicting key kinetic rate parameters of heterogeneous elementary steps that dictate the mechanistic branching of electrosynthesis reactions.

As the starting foundation for this perspective article, we refer to the large body of work by Savéant and co-workers on electrochemical reaction mechanisms as found in their numerous seminal publications,^17–21^ and textbook.^22^ In particular, we focus on electrosynthesis reactions proceeding through the common electrochemical-chemical-electrochemical or “E-C-E” mechanism. Note that this could also encompass reactions in which there are subsequent mechanistic steps (e.g., “E-C-E-C”), as long as the initial part of the reaction follows the “E-C-E” mechanism. In this Perspective, we choose to focus on anodic (oxidative) reactions, but much of the discussion will generally apply to cathodic (reductive) processes as well. The general form of an anodic “E-C-E” electrosynthesis reaction occurring via direct electrolysis is^22^

1 $$A\overset{k_{\mathrm{ET}}}{⇌}B+e^{-}$$

2 $$B\overset{k_{r}}{⇌}C$$

3 $$C\overset{k_{\mathrm{ET}}}{⇌}D+e^{-}$$

Following the initial oxidation of substrate “A” at the anode, the reactive intermediate (radical or cation radical) “B” undergoes a chemical reaction to “C”, which then undergoes subsequent oxidation. Due to the generality of such electrochemical processes, we will further restrict the scope to reaction mechanisms in which the “E” steps correspond to “outer-sphere” electron transfer at inert electrodes, and not “inner-sphere” processes in which electron transfer is coupled to chemisorption on the electrode surface. We are thus excluding discussion of “electrocatalytic” processes in which chemisorption and catalysis on the electrode surface are an essential part of the reaction mechanism. While many of the discussed paradigms (e.g., electrical double layer effects) are indeed also relevant to electrocatalysis processes, the focus on direct electrolysis “E-C-E” reactions with “outer-sphere” electron transfer puts particular emphasis on radical ion reaction kinetics as well as solvophobic forces dictating substrate residence time near the electrode, as will be discussed in detail throughout this article.

Returning to eqs 1–3, the simple notation of these mechanistic steps obscures the full mechanistic detail of the electrosynthesis reaction that could be crucial for interpreting or optimizing the overall reaction yield/selectivity. Specifically, it is well-known that electrochemical reactions of this form can either occur via the formal E-C-E pathway in which the second electron transfer step occurs heterogeneously at the electrode interface, or instead through a disproportionation (henceforth “DISP”) mechanism, in which the second electron transfer occurs within the bulk solution (away from the electrode), via homogeneous electron transfer usually involving species B and C, i.e.^22^

4 $$B+C\overset{k_{\mathrm{ET}}}{⇌}A+D$$

Seminal work by the Savéant group,^23–27^ and others,^28,29^ resulted in criteria for distinguishing between E-C-E and DISP pathways based on electroanalytical techniques (and criteria for when such distinction is possible). Determining whether an electrosynthesis process proceeds via the E-C-E or DISP pathways is important in cases where the distinct pathway dictates the overall reaction yield/selectivity. Often, the rate of the second electron transfer step (in either eqs 2 or 4) is critical for target product yield,^30^ as it governs the lifetime of radical intermediates that may undergo a variety of undesired side reactions. For E-C-E pathways, the second electron transfer is typically expected to be fast given that the working electrode is usually at an overpotential (i.e., lower oxidation potential for the second compared to initial oxidation), while for DISP pathways the second electron transfer proceeds via second-order kinetics (eq 4) in concentrations of electrogenerated species, and may be slower. An additional difference is that for the E-C-E process, all mechanistic steps of the reaction occur within the electrochemical double layer (EDL), formed at the electrode/electrolyte interface, as a consequence of the fact that electron transfer is typically a short-ranged process corresponding to distances ≤1 nm.^31^ Thus, in the E-C-E process, the kinetic rate constants dictating reaction yield/selectivity will be modulated by the EDL environment, whereas for the DISP process, the selectivity-determining steps will have rate constants corresponding to the homogeneous reaction kinetics.

There is much physical basis and indeed many reported empirical examples for how reaction rates may be substantially modulated by the EDL environment at an electrode/electrolyte interface. Large electric fields at the electrode (originating from the applied voltage) alter adsorption/binding to the electrode surface,^32^ and may impart “electrostatic catalysis” by stabilizing transition states/intermediates and thus impacting reaction kinetics.^33–38^ Furthermore, the concentrated ionic environment coupled with low dielectric screening of interfacial solvent molecules,^39–42^ entails that reaction kinetics are often strongly modulated by the electrolyte ions that accumulate within the double layer;^43–50^ correspondingly protonation/deprotonation kinetics is impacted by altered activities of reaction intermediates.^51^ In addition, the solid electrode surface may sterically control product yield for reactive substrates that are adsorbed or closely packed against the electrode surface.^52,53^ It is additionally well-known that electron transfer rates differ within the electrostatic environment of the electrode/electrolyte interface.^42,54–56^ In synthetic organic electrochemistry, there are many examples of how reaction selectivity/yield is altered by changes in the electrochemical environment.^1^ A prototypical example is the electrohydrodimerization of acrylonitrile, which is an industrial process that was originally developed by Baizer.^12,13,57,58^ This electrosynthesis proceeds with a poor yield of the intended adiponitrile product when alkali cation-based electrolytes are utilized, but the yield is dramatically improved when tetraalkylammonium cation-based electrolytes are utilized instead. Clearly in this case, the nature of the EDL environment plays a pronounced role in modulating the kinetics of both the target and competing reaction pathways involving the radical and radical ion intermediates.^44,45^

Computational electrosynthesis research thus seeks to elucidate how the reaction yield and selectivity are dictated by the kinetics of the elementary mechanistic steps of both the target reaction and competing side reactions and how the relevant rate constants are altered/modulated by the EDL environment at the working electrode/electrolyte interface. The goal is to utilize the developed mechanistic understanding to effectively design/optimize the electrochemical reaction conditions for improved yield/selectivity of existing electrosynthesis processes and/or to develop novel electrosynthesis processes that would otherwise be inefficient/unachievable without such optimization of reaction conditions. While computational studies are clearly also important for characterizing homogeneous reaction kinetics/rate constants, the particular focus here is on heterogeneous reaction kinetics/rate constants for processes occurring at the electrode/electrolyte interface. The motivation for this focus is that (1) computational approaches are well-poised to elucidate the atomistic structure/interactions within the EDL and subsequent modulation of reaction kinetics at length scales/time scales that are difficult to probe experimentally; and (2) the heterogeneous reaction kinetics/mechanisms at an electrode/electrolyte interface are particularly compelling due to the mentioned possibilities of altered reaction rates from the strongly perturbing interfacial/electrostatic environment. We note that while there has been substantial progress in direct experimental characterization of chemical processes at electrochemical interfaces,^59–66^ experimental work is not extensively discussed since our focus is on computational approaches.

So are there “rules of thumb” for predicting when an electrochemical reaction might proceed via the fully heterogeneous E-C-E pathway (vs DISP)? Savéant and Costentin formulate an expression for the thickness of the reaction layer “μ” (e.g., spatial region where electrochemical reaction is occurring), based on consideration of the rate constant “*k*_C_” of the chemical step (“C” in E-C-E) and mass transport of the substrate described by diffusion coefficient “*D*”^22^

5 $$\mu =\sqrt{\frac{D}{k_{C}}}$$

Note that we use the notation “*k*_C_ = *k*_r_” for the chemical rate constant to be consistent with the notation utilized in Section 3. Considering that typical values of the diffusion coefficient are on the order *D* ∼ 10^–5^ cm^2^/s, it is thus estimated that the reaction layer thickness μ will be of the same length scale as the EDL (e.g., ∼1 nm) only for very fast reactions, with rate constants of order *k*_C_ ∼ 10^10^ s^–1^ (i.e., either first or pseudo-first-order).^17^ According to these authors, “It is only for such fast reactions that their kinetics may be perturbed by the strong electric field present in the close vicinity of the electrode.”^22^ However, the estimate provided by eq 5 may be incorrect in many cases, as it omits consideration of strong solvophobic forces that can lead to longer residence times near the electrode surface. For example, we will present simulation data in this manuscript that suggests that because of relatively slow desorption from the electrode surface (i.e., solvophobic forces), reactions with rate constants of *k*_C_ ∼ 10^7^–10^8^ s^–1^ (or smaller) may proceed though heterogeneous E-C-E mechanisms, and thus be modulated by the EDL environment (i.e., *k*_C_ orders of magnitude smaller than the eq 5 estimate). For reactive, electrogenerated radical/ion intermediates, rate constants of this magnitude are common.^67–69^

This Perspective is organized as follows. In Section 2, we discuss the construction of atomistic computational models of the electrochemical cell under working conditions, and approximations associated with replicating the experimental conditions. This involves a discussion of how the surface charge on the working electrode should be set to best model the electrical double layer environment at the working electrode under experimental electrolysis conditions. In Section 3, we discuss how solvophobic forces dictate substrate/intermediate residence times at the working electrode interface, which is an important kinetic parameter dictating branching between heterogeneous “E-C-E” and homogeneous “DISP” reaction pathways. We discuss how classical molecular dynamics simulations are used to compute free energy profiles for substrate/intermediate association/adsorption to the working electrode surface at applied potential and present case studies illustrating how this adsorption process is modulated by solvent, electrode charge, and nature of the organic substrate. We then discuss how desorption rate constants are computed from direct dynamics simulations, providing one of the key rate parameters for the mechanistic determination. Finally, in Section 4, we discuss computations of reaction free energies of cation radical intermediates within the EDL environment modeled fully at atomistic resolution. All computations utilize hybrid quantum mechanics/molecular mechanics (QM/MM) MD simulations with free energy sampling, and we give a brief overview of this method as applied to electrosynthesis research. We then discuss various case studies of anodic intramolecular coupling reactions in Sections 4.1 and 4.3, benzyl alcohol oxidation in Section 4.2, and anodic olefin electrolysis in water-modulated ionic liquid environments in Section 4.4. In these case studies, we explore questions of how reaction free energy barriers are modulated by the nature of the electrochemical double layer (EDL) at the electrode/electrolyte interface. Overall, the focus is on computations of kinetic parameters for heterogeneous elementary reaction steps that dictate mechanistic branching and thus product selectivity/yield, and elucidating the influence of electrochemical reaction conditions.

Inevitably, there are numerous important computational electrochemistry topics that we deliberately do not discuss in this perspective article due to both space constraints and/or the existence of excellent reviews/discussions elsewhere. These include: the first-principles computation of redox potentials;^70–72^ computation of heterogeneous electron transfer rates for either “outer-sphere” ^73–81^ or “inner-sphere” ^82–84^ processes; the theory/computation of proton coupled electron transfer (PCET) processes;^85–87^ computational electrosynthesis studies with focus on electrode surface catalysis;^37,88–90^ homogeneous reactions with electrochemical mediators;^91,92^ ab initio modeling of metal electrode/aqueous interfaces with either explicit or implicit solvent description;^93–103^ ab initio Pourbaix diagrams of electrode surfaces or molecular electrocatalysts;^87,104–108^ first-principles prediction of voltammograms;^109–111^ classical electrostatic, constant potential methods as applied to predicting electrical double layer structure and/or capacitance.^112–122^ We note that some of these topics have been discussed in a recent series of review articles on computational electrochemistry published within Chemical Reviews.^123^

## 2 Computational Modeling of the Electrochemical Cell under Working Conditions

We discuss the computational considerations and methods for modeling the electrochemical cell under working conditions of a target electrosynthesis process. Because the goal is to explicitly study how the atomistic structure/interactions within the EDL environment impact the kinetics of the relevant microscopic reaction steps, we restrict our focus to computational approaches consisting of (1) classical molecular dynamics (MD) simulations for physical processes that do not involve chemical bond breaking/forming; and (2) hybrid quantum mechanics/molecular mechanics (QM/MM) MD simulations to investigate the chemical steps of the mechanism. The choice of methods is based on the study of E-C-E electrosynthesis processes involving “outer-sphere” electron transfer at an inert electrode, and not “inner-sphere” processes in which electron transfer is coupled to chemisorption on the electrode surface (i.e., not “electrocatalytic” processes). In this case, the electrode can be modeled at the molecular mechanics or force field level, as the chemical steps involve only the solvated substrate/intermediates. QM/MM represents a computationally efficient choice for the multiscale modeling of such reactions within the complex electrochemical environment described at fully atomistic resolution.^124^

The starting point is then to “build” an atomistic model of the electrochemical cell, consisting of electrodes and electrolyte and incorporating the applied external potential (at working conditions). It is useful here to consider an example electrosynthesis reaction for explicit discussion, and in this section, we consider the anodic oxidation of a benzyl alcohol substrate to its corresponding aldehyde. Figure 1 shows the proposed “E-C-E-C” mechanism for the anodic process in which the two electrochemical steps consist of outer-sphere electron transfer, and the chemical steps are deprotonation reactions of the cation radical and cation intermediates.

**Figure 1:**
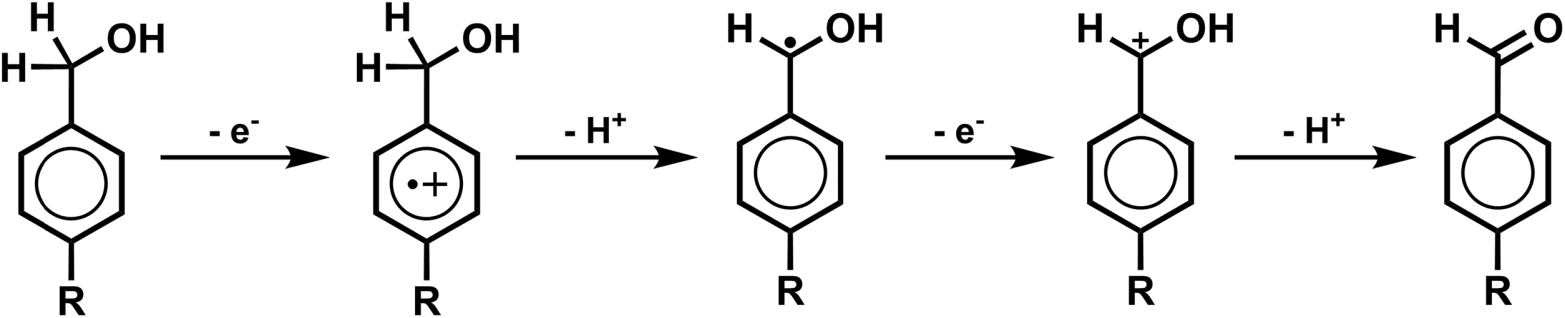
Schematic depicting the “E-C-E-C” reaction steps and intermediates for anodic electrolysis of a benzyl alcohol substrate to its corresponding aldehyde.

To model the various steps of this electrochemical process within the electrochemical cell, specific electrodes and electrolytes must be chosen. For this case, the simulated system is based on experimental work of Wang et al. that utilized direct electrolysis of benzyl alcohols to synthesize the target aldehydes.^125^ These authors employed a carbon anode, nickel plate cathode, water/acetonitrile solvent mixture, and various electrolyte choices, including tetrabutylammonium tetrafluoroborate (Bu_4_NBF_4_), lithium perchlorate (LiClO_4_), as well as other electrolytes. Constant current electrolysis was utilized, and within a flow cell. Based on these experimental reaction conditions, simplifications are made to conduct the computer simulations. Because the microstructure of experimental carbon electrodes such as glassy carbon or reticulated vitreous carbon (RVC) is unknown/difficult to model, carbon electrodes are modeled as the basal plane of graphite (i.e., a graphene surface). The goal here is that the planar graphene surface may provide a good approximation of the ∼1–2 nm length scale comprising the inner layer of the EDL, although micron-scale phenomena (surface area, mass transport) at the experimental carbon electrode would clearly not be captured. Also, identical graphite electrodes are chosen as the anode and cathode for the simulation, despite a nickel plate cathode being used in the experiment. In the experiment, the hydrogen evolution reaction (HER) is the counter cathodic process to the anodic reaction (Figure 1), which is the motivation for the catalytic nickel plate cathode, but since the cathodic HER is not being modeled in the simulation, the type of cathode in the simulation is immaterial.

Other simplifications/approximations are required, as well. Constant current electrolysis is the most commonly utilized condition in preparative electrosynthesis to facilitate maximal product conversion.^1,3,5,6^ Mechanistic understanding of constant current electrolysis relies on the determination of electrode roughness factors, to relate the actual vs geometric current density and thus applied overpotential, which in cases has led to inconsistent interpretation.^53^ For computational studies, a reasonable approach then is to simulate constant voltage conditions, realizing that the derived results/interpretation will vary with the precise overpotential during constant current electrolysis. Then, how should the applied (fixed) voltage be modeled within the classical/molecular mechanics treatment of the electrodes in the simulations? There exist myriad algorithms for employing constant potential/fixed voltage conditions within MD simulations,^112–122,126^ and any of these approaches certainly could be utilized. However, a crucial issue/concern is the capacitance of the electrochemical interface, since both the capacitance and applied voltage determine the net charge on the electrode(s) and net charge of the EDL (the latter as required by charge neutrality). If the goal is to model the atomistic structure and interactions within the EDL (and subsequent influence on reaction rates), the absolute charge on the electrode, which dictates the net charge within the EDL should be determined as accurately as possible. Unfortunately, classical constant potential/fixed voltage MD methods incorrectly predict the magnitude of capacitance, often by a severe amount, due to the neglect of the “electronic, surface-dipole contribution”.^127^ This means that while constant potential/fixed voltage approaches will capture explicit image charge interactions, they may be simulating a double layer that is often undercharged by up to a factor of 2 or more if the simulation voltage is set to the experimental working potential (i.e., capacitance is underestimated in simulation).^127^ We note that more recent constant potential MD algorithms have been developed to attempt to address this problem.^117^

Figure 2 shows a depiction of the potential profile across an electrochemical cell to distinguish between the experimental voltage drop and the corresponding simulation voltage drop for the electrodes modeled classically, as described above. The experimental voltage drop between the electrodes corresponds to the difference of their absolute potentials (using eV units to relate energies and potentials),^129^ or Δ*V*_exp_ = −μ̃_e_^anode^ + μ̃_e_^cathode^, where μ̃_e_^electrode^ is the electrochemical potential of the electrode. The electrochemical potential is equivalent to the Fermi level of the electrode, and is given as μ̃_e_^electrode^ = μ_e_^electrode^ – ϕ_electrode_, where μ_e_^electrode^ is the electron chemical potential of the electrode material, and ϕ_electrode_ is the inner potential of the electrode (e.g., electrostatic potential within the phase) as depicted in Figure 2. For simplicity, we assume the two electrodes consist of the same material, so that their chemical potentials are the same (μ_e_^anode^ = μ_e_^cathode^), such that the experimental voltage drop then corresponds to the difference of the inner potentials of the electrodes, Δ*V*_exp_ = ϕ_anode_ – ϕ_cathode_.^96^ As depicted in Figure 2, this difference ϕ_anode_ – ϕ_cathode_ has a contribution from the surface potentials, χ_anode_, χ_cathode_, that arise from the “electron spillover” or “electronic surface dipole” at the electrode surface.^128^ This electronic surface dipole contribution is precisely what is omitted in (most) classical fixed voltage/constant potential methods. Hence, the appropriate voltage difference in a corresponding classical simulation is Δ*V*_sim_ = ψ_anode_ – ψ_cathode_, where ψ_anode_, ψ_cathode_ are the outer potentials, at the exterior of the electrode surface. It is important to note that setting the simulation voltage drop to the experimental voltage drop Δ*V*_sim_ = Δ*V*_exp_ = ϕ_anode_ – ϕ_cathode_ would result in an incorrect description of the EDL potential profile, since the simulation does not contain the surface potential terms, χ_anode_, χ_cathode_.

**Figure 2:**
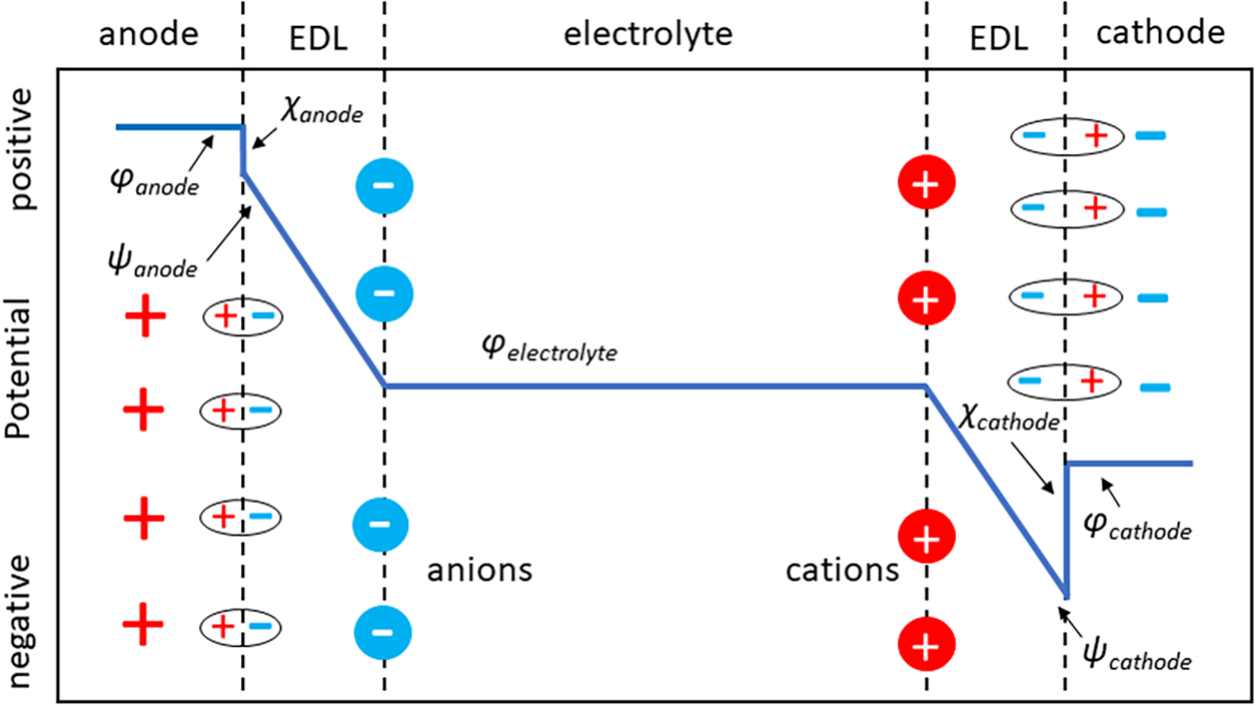
Potential profile across electrochemical cell, depicting the inner ϕ, outer ψ, and surface χ potentials at each electrode; these are related by χ = ϕ – ψ. The ovals with ± charge depict the surface dipole of the electrode, formed from “electron spillover” off the ionic nuclei of electrode atoms at the surface; these are greatly exaggerated in size for visual clarity. Note that the surface potential is larger at the negatively charged cathode compared to the positively charged anode, as depicted by relative size of surface dipoles; this corresponds to the electronic surface term giving a negative contribution to the capacitance of the electrode/electrolyte interface.^128^

How should the two-electrode cell in the simulation be mapped to a typical three-electrode cell in an experimental setup with working, reference, and auxiliary/counter electrodes? Assuming the anode is the working electrode (i.e., anodic electrosynthesis), the second electrode (cathode) in the simulation is best thought of as the auxiliary (and not reference) electrode in an experimental three-electrode setup. The voltage profile of the auxiliary electrode is immaterial in an experiment (at least regarding the anodic process), and as such, the voltage profile of the cathode in the simulation setup is immaterial. In the simulation, there is no need for a reference electrode, as the natural reference in the simulation is the potential of zero charge (PZC) of the working electrode. The electrode charge is trivially set/determined in the simulation, and the potential drop ψ_anode_ – ϕ_electrolyte_ between the working electrode and the electrolyte can be determined at the zero charge state by computing the Poisson profile.^127^ As the working electrode is charged, the potential drop ψ_anode_ – ϕ_electrolyte_ will change and can be reported relative to the PZC value. However, comparing this simulation potential against a corresponding experimental potential (reported relative to PZC) would be erroneous due to the omission of the surface potential χ_anode_ in the simulation, which is voltage dependent (and typically unknown).^128^

To resolve the discrepancy discussed above, we choose to model the working electrode with a uniform, constant charge during the simulation, as fixed to the experimental working electrode surface charge. Note that if the working electrode is charged to the corresponding experimental surface charge, the simulated ψ_anode_ – ϕ_electrolyte_ will match the experiment, and the anodic double layer will be simulated at the correct net charge. The surface charge on the electrode(s) is thus determined as σ = *C*_exp_Δ*V*_PZC_, where σ is the surface charge density on the electrode, *C*_exp_ is the experimentally determined capacitance of the working electrode/electrolyte interface, and Δ*V*_PZC_ is the working potential defined relative to the potential of zero charge (PZC) of the electrode. Determining the charge σ to set on the electrodes thus consists of determining values *C*_exp_ and Δ*V*_PZC_. For Δ*V*_PZC_, the PZC of the electrode/electrolyte interface must be known and estimated as well as the initial oxidation potential of the substrate. Figure 3 shows an example diagram used to determine Δ*V*_PZC_ for the specific electrolysis of *para*-methoxybenzyl alcohol (Figure 1) with a carbon working electrode. Literature values for the substrate oxidation potential (1.3 V vs Ag/AgNO_3_)^130^ and electrode PZC (−0.2 V vs Fc/Fc^+^)^131^ must be converted to a common reference scale (SCE), which results in an estimate of Δ*V*_PZC_ = 1.46 V (Figure 3). Computational predictions of redox potentials^70–72^ may be used to appropriately adjust Δ*V*_PZC_ for related substrates, in lieu of experimental data.

**Figure 3:**
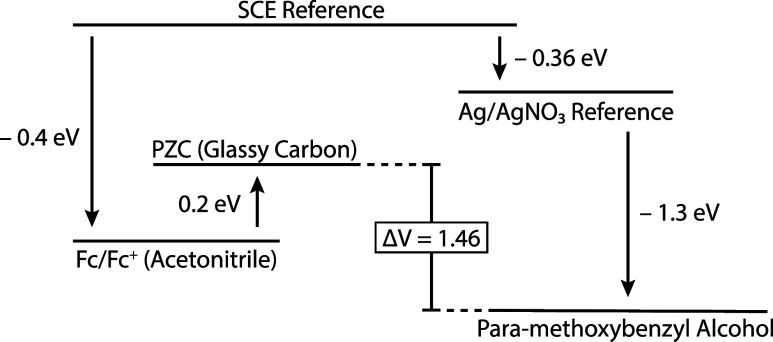
Energy level diagram indicating the relation of *para*-methoxybenzyl alcohol oxidation potential to glassy carbon electrode PZC, and relative to common reference potentials. The arrows/line spacing are not drawn to scale, but rather, spacing is chosen for readability. Also note that arrows denote relative energy, which is opposite in sign to the relative voltage.

The largest uncertainty in determining the electrode surface charge σ under working conditions comes from estimating the capacitance, *C*_exp_. Typically, the electrolyte ion concentration (≥0.1 M) and working potential (Δ*V*_PZC_ ≥ 1 V) are both large enough that only the inner/Helmholtz region of the double layer contributes to the capacitance (i.e., no contribution from the diffuse or Gouy–Chapman region).^132^ However, values for the differential capacitance of the inner/Helmholtz layer can dramatically vary with electrode material/crystallographic orientation and solvent and electrolyte composition; for example values range from *C*_exp_ ∼ 5–15 μF/cm^2^ for concentrated organic electrolytes/ionic liquids at carbon electrodes,^133–137^ to *C*_exp_ ∼ 20–60 μF/cm^2^ at metal/aqueous electrochemical interfaces.^31,128,138–140^ Furthermore, the differential capacitance usually exhibits substantial voltage dependence, which has been neglected for simplicity in the above approximation: σ = *C*_exp_Δ*V*_PZC_. Because of such variations, it would be ideal if the surface charge and/or capacitance were experimentally measured for the specific electrochemical cell and working potential(s) utilized in the target electrosynthesis experiment. If this is not the case, estimates must be made, and if possible, simulations can be run at systematically varying σ to identify trends/dependence on overpotential, while indicating the sensitivity of predictions to uncertainty in *C*_exp_.

Once the charge at working potential is estimated (e.g., σ ∼ 14.6 μC/cm^2^ = 10 μF/cm^2^·1.46 V for Figure 3), the electrodes are then uniformly charged to this value ± σ for anode/cathode. The electrostatics of the system is then treated using 3D-Ewald sums (or more specifically, particle mesh Ewald),^141^ which introduces (artificial) periodicity along the dimension of the electrochemical cell. To avoid electrostatic interactions between periodic replicas of the cell along this dimension, a large vacuum gap (typically 10–20 nm) is added to the system to separate the electrodes in the principal cell from images of the electrodes in the periodic replica cells. For this simulation construct, it should be checked whether or not the “dipole correction” proposed by Yeh and Berkowitz is significant, and included if it is;^142^ in our experience, this correction is usually small and can be neglected for a sufficiently large vacuum gap. We note that the major limitation of the “constant electrode charge” approach described above is the neglect of explicit image charge interactions that may affect the interfacial free energy profiles of charged reactive intermediates. Incorporating such image charge interactions (while preserving accurate capacitance values^117^) would be an important goal for future work.

A snapshot of the constructed electrochemical cell with graphite electrodes, water solvent, and a 0.4 M LiClO_4_ electrolyte is shown in Figure 4. The electrode structures are built utilizing the Atomic Simulation Environment (ASE) software.^143^ We utilize the OPLS-AA force field for solvent molecules (in this case, SPC/E water model),^144^ with custom parameters for electrolyte ions,^145–147^ and electrode atoms.^148^ We target an electrochemical cell size of roughly ∼10 nm separating the anode/cathode, which gives a sufficient bulk electrolyte region of ∼5–6 nm separating the EDLs of the two electrodes; the number of solvent/electrolyte molecules/ions is then chosen based on this target system size. The initial configuration for the solvent/electrolyte is created with the PackMol software.^149^ In our experience, complex electrolyte systems must be prepared with PackMol at substantially lower density than equilibrium to avoid high-energy initial conditions (which would otherwise crash the simulations). This means that the density of the electrochemical cell must be subsequently equilibrated. To do this, we employ a hybrid Monte Carlo/molecular dynamics (MC/MD) scheme utilizing the OpenMM software package,^150^ in which MD moves of electrolyte positions are interspersed with MC translational moves of one electrode along the dimension of the electrochemical cell.^119^ In this way, the electrode remains rigid while adjusting its position to equilibrate the density of the liquid solvent/electrolyte. A final subtle point about the simulation construction concerns the bulk ionic concentration after equilibration once the electrodes are charged to their working potential. Because of the finite system size, ionic charging of the double layers depletes ion concentration from the bulk, the extent of which depends on the specific system size, concentration, charge, etc., but can be significant. This means that the reported “bulk” ion concentration of the simulation should not correspond to what was initially prepared, but rather from analyzing the ion density profile in the bulk electrolyte region of the system following equilibration to the working electrode charge.

**Figure 4:**
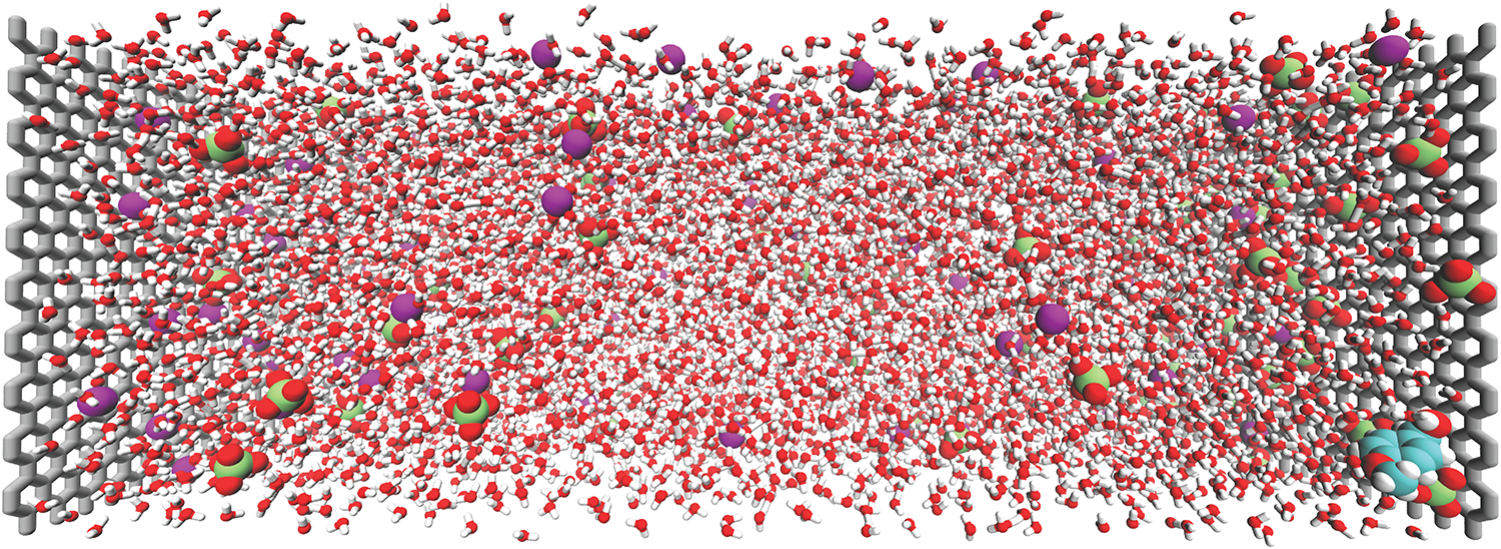
Example construction of an electrochemical simulation cell used in classical and QM/MM molecular dynamics simulations. The system shown consists of two graphite electrodes with a 0.4 M LiClO_4_ aqueous electrolyte. Reproduced with permission from ref 151. Copyright 2025 American Chemical Society.

## 3 Solvophobic Forces Modulate the E-C-E vs DISP Pathway: Computing Substrate/Electrode Association Free Energies and Corresponding Desorption Rate Constants

As previously mentioned, whether the steps of an electrosynthesis reaction mechanism proceed heterogeneously via formal E-C-E or homogeneously via the DISP pathway may have significant consequences for the overall reaction yield and selectivity. In the case of the benzylic alcohol electrolysis (Figure 1), a DISP mechanism for the second electron transfer entails that the benzyl radical intermediate may be susceptible to homogeneous side reactions, given the slower second-order kinetics governing the homogeneous electron transfer. In contrast, for the E-C-E pathway, the second heterogeneous electron transfer is expected to be relatively fast given that the oxidation potential of the radical intermediate is typically lower than that of the initial substrate (i.e., substantial overpotential for the second electron transfer); thus, for E-C-E, the benzyl radical intermediate may be relatively short-lived and less susceptible to side reactions.

Figure 5 shows a schematic depiction of the mechanistic steps and associated rate constants governing the E-C-E versus DISP pathways. The elementary steps (1–4) and labels of intermediates “B”, “C”, “D” in Figure 5 correspond to those of eqs 1–4 in Section 1. Initially, the substrate “A” must be at the working electrode surface (e.g., within ∼1 nm distance), to allow for the initial heterogeneous electron transfer with rate constant *k*_ET,1_^het^. After oxidation/electron transfer to generate reactive intermediate “B” (typically a radical or cation radical), the intermediate can either remain at the working electrode to undergo subsequent chemical and second electron transfer steps heterogeneously, or “desorb” from the working electrode surface and out of the inner double layer, and eventually migrate to the bulk to undergo the remaining steps homogeneously. The terminology “desorb” rather than “diffuse” is intentional and an important distinction. Here, we are not referring to typical mass transport as governed by Fick’s law. Rather, we are referring to the substrate/intermediate “desorbing” from a well-defined free energy minimum at a location within the inner double layer and proximal to the electrode surface that arises from strong solvophobic forces. Note that the terminology “desorb” is not meant to imply “chemisorption”, as our entire discussion is focused on systems where chemisorption does not occur (inert carbon electrodes). This will become clear in the following discussion.

**Figure 5:**
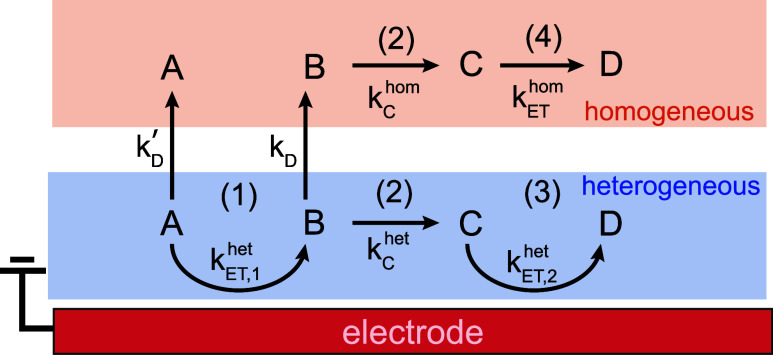
Schematic of possible elementary steps and corresponding rate constants for an electrochemical transformation of reactant/substrate “A” proceeding via the heterogeneous E-C-E or homogeneous DISP pathways. Labels of intermediates “B”, “C”, “D” and elementary steps (1–4) correspond to those of eqs 1–4. Rate constants correspond to elementary steps of: electron transfer, either heterogeneous “*k*_ET_^het^”, or homogeneous/disproportionation “*k*_ET_^hom^”; chemical steps, either heterogeneous “*k*_C_^het^” or homogeneous “*k*_C_^hom^”; desorption of the substrate/intermediates from the inner double layer region near the electrode surface, with rate constants “*k*_D_^′^” and “*k*_D_” for the neutral substrate “A” and cation radical intermediate “B” respectively.

For the simplest case that all rate constants correspond to first-order or pseudo-first-order rate laws, whether the reaction goes through the E-C-E or DISP pathway will clearly depend on the relative magnitudes of the “desorption” rate constants *k*_D_ of the intermediate(s), chemical rate constant *k*_C_ (which could differ in the heterogeneous *k*_C_^het^ or homogeneous *k*_C_^hom^ environment), and heterogeneous electron transfer rate constant for the intermediate *k*_ET,2_^het^. We will shortly discuss quantitative values for *k*_D_ as computed from MD simulations, and subsequently discuss *k*_C_^het^ values computed from transition state theory for a few example electrosynthesis reactions. It is initially useful to provide example reference values to give a sense of the relevant kinetic time scales involved. Fast heterogeneous electron transfer corresponds to the case when there is minimal distortion of the substrate molecular geometry following electron transfer (this corresponds to a small “inner-sphere” reorganization energy, and we note that the “outer-sphere” reorganization energy near an electrode is also typically small^31,42,152^ ). An example is heterogeneous electron transfer for the ferrocene/ferrocenium redox couple with a standard rate constant of *k*_ET_^het,0^ ∼ 10–40 cm/s. This can be converted to an estimate of *k*_ET_^het,0^ ∼ 10^9^ s^–1^, based on the expected ET reaction distance.^75,153,154^ The rate constant *k*_ET,2_^het^ for the second electron transfer step in Figure 5 is likely much larger given the expected dependence on overpotential, e.g., *k*_ET,2_^het^ = *k*_ET,2_^het,0^ e^(1−α)*F*(*E*–*E* ^0^)/*RT*^, where *E* – *E* ^0^ is the overpotential, α is the transfer coefficient, and F is Faraday’s constant.^31^ As mentioned, it is typically the case that the oxidation potential of the radical intermediate “C” is lower than that of the initial substrate “A”, so there is a substantial overpotential for the second electron transfer at the working electrode potential.

For estimates of the rate constant of the chemical step, *k*_C_, we focus on the relatively common case in which the reactive intermediate “B” is a cation radical. Cation radical intermediates often react via either first-order kinetics, e.g., intramolecular cyclization, or pseudo-first-order kinetics, e.g., nucleophilic attack or deprotonation by the solvent (water, methanol, etc.).^67–69,155^ As an example, cation radicals formed from oxidation of olefin groups often have very short (e.g., nanosecond) lifetimes, with reaction rate constants of *k*_C_ ∼ 10^9^–10^10^ s^–1^.^67–69^ On the opposite end of the spectrum, cation radicals formed from oxidation of highly conjugated aromatics are less reactive, for example diphenylanthracene cation radicals exhibit much smaller rate constants for solvent nucleophilic attack, e.g., *k*_C_ ∼ 10^0^–10^2^ s^–1^;^155^ electrochemical reactions of this latter substrate class most likely proceed via the DISP pathway due to the slow chemical step.^155^ In this discussion, we have not distinguished between the heterogeneous *k*_C_^het^ or homogeneous *k*_C_^hom^ reaction pathways; some examples for this distinction will be provided in Section 4.

The above estimates provide a “rule of thumb” for when E-C-E or DISP mechanisms may be expected in the case of fast electrical/chemical steps. Figure 5 shows that the heterogeneous E-C-E mechanism is likely if “desorption” of the intermediate(s) from the working electrode surface is slower than the electrical/chemical steps. For example, if a hypothetical electrochemical reaction has *k*_ET,2_^het^, *k*_C_^het^ ∼ 10^9^ s^–1^, then a desorption rate constant *k*_D_ < 10^9^ s^–1^ would likely lead to the E-C-E mechanism. Note that in this case, the relevant desorption rate constant *k*_D_ is for the cation radical intermediate “B”; the question then becomes, what are the residence times of cation radical intermediates near the working anode, which has a substantial positive charge? Based on electrostatic arguments, one may think that cation radical intermediates would immediately be repelled from the positive charge of the working anode. In contrast, however, for moderate electrode surface charge, cation radical intermediates often exhibit favorable association free energies within the anodic double layer near the electrode surface, due to both strong solvophobic forces and electrostatic attraction with anions in the EDL.

Molecular dynamics simulation provides a straightforward approach to compute the desorption rate constants, *k*_D_, for electrogenerated intermediates near the working electrode. Related to computing *k*_D_, MD simulations can also be used to compute free energy profiles for the intermediate(s) as a function of distance from the working electrode. We first discuss such free energy profiles, or potentials of mean force (PMFs), and then subsequently discuss the desorption rate constants *k*_D_. Although the key kinetic parameter *k*_D_ is that of the intermediate “B” (Figure 5), we first discuss the desorption free energy profiles for the neutral substrate, as this provides an insightful comparison. Figure 6 shows PMFs for *p*-methoxybenzyl alcohol as an example substrate for the electrosynthesis reaction in Figure 1. These are computed as a function of the distance of the benzyl ring from the working carbon anode (graphite) as a function of electrode surface charge. Figure 6a shows PMFs computed within water solvent with 0.4 M LiClO_4_ electrolyte, and Figure 6b shows PMFs computed within methanol solvent with 0.4 M LiClO_4_ electrolyte. These PMFs were computed utilizing standard umbrella sampling techniques, with 18 evenly spaced umbrella windows spanning the electrode separation distance range and 20 ns of sampling per window. The weighted histogram analysis method (WHAM) was subsequently used to reverse-bias the sampled distributions and generate the PMFs.^156^ We note that analogous PMFs could also be generated from direct (unbiased) MD simulations used to compute desorption rate constants *k*_D_ (discussed subsequently), although this typically requires longer simulation times for adequate sampling. In this work, we restrict our discussion to carbon electrodes; PMFs for similar organic substrates at metal electrodes have been reported in the literature.^157^

**Figure 6:**
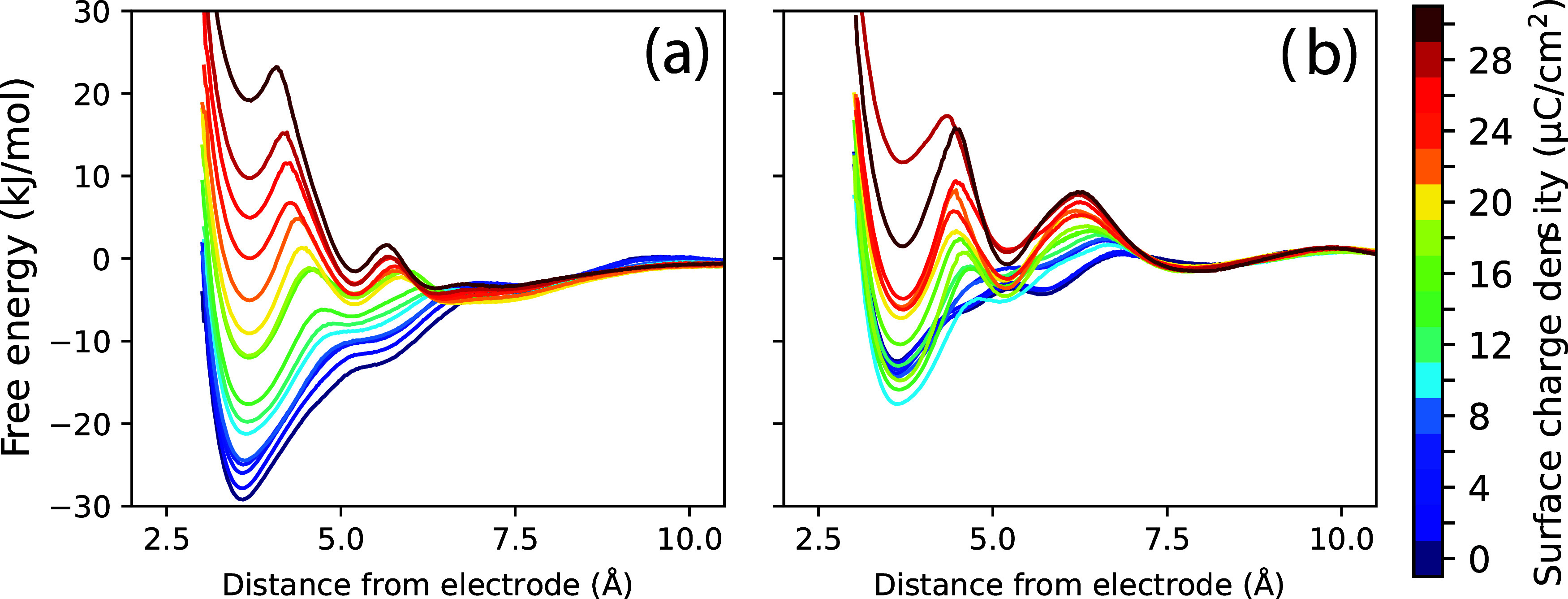
Free energy profiles (PMFs) for *p*-methoxybenzyl alcohol near the working carbon anode as a function of surface charge. (a) Water solvent with 0.4 M LiClO_4_ electrolyte and (b) methanol solvent with a 0.4 M LiClO_4_ electrolyte. (a) is adapted with permission from ref 151. Copyright 2025 American Chemical Society.

The immediate realization is that there are pronounced minima (and corresponding barriers) in the PMFs, which arise from solvophobic forces within the electrolyte. It is important to note that the free energy minima are not primarily a result of the direct benzyl alcohol/graphite interaction but rather are mediated indirectly by the solvent/electrolyte. This is clear from comparing the PMFs computed for the water (Figure 6a) and methanol (Figure 6b) based electolytes; the free energy minima are significantly lower in energy for the water-based electrolyte, due to the larger solvophobic (hydrophobic) forces. It is also evident that the PMFs are substantially modulated by the electrode charge. The estimated working charge of the electrode is σ ∼ 14.6 μC/cm^2^ = 10 μF/cm^2^·1.46 V (i.e., σ = *C*_exp_Δ*V*_PZC_), and PMF profiles in Figure 6 span the range 0 ≤ σ ≤ 30 μC/cm^2^ of electrode surface charge. The green curve(s) in Figure 6 thus correspond to the approximate working electrode potential (i.e., no overpotential). At the working potential, the free energy minimum near the electrode is ∼−10 to −20 kJ/mol for the water-based electrolyte, and ∼−5 to −10 kJ/mol for the methanol-based electolyte, relative to the bulk; thus there is a significant substrate association with the working electrode. Note that these free energy minima are most substantial when the electrode is uncharged (PZC), which corresponds to the dark blue PMF curve. As the electrode is charged, anions from the electrolyte accumulate to a substantial concentration within the EDL and solvent molecules within the inner layer(s) become highly structured, which diminishes the solvophobic attraction of the substrate with the working electrode surface. Eventually, when the electrode is highly charged (large overpotential), the substrate may be excluded from direct contact with the surface due to the highly concentrated ions and structured solvent molecules within the EDL; similar conclusions have been made from computational studies of cathodic reduction of related benzyl bromide substrates.^158^

The PMFs in Figure 6 have multiple minima, which correspond to different configurations of the *p*-methoxybenzyl alcohol substrate near the working electrode surface. These configuration motifs are shown in Figure 7, as depicted from molecular dynamics snapshots of the working electrode surface. Figure 7 panels a–c depict structural motifs of the three minima in the PMFs (Figure 6), respectively, spanning the closest (∼3.5 Å) to the farthest (∼7.5 Å) distance from the electrode surface. The closest minimum, which is the most stable at low-to-moderate electrode charge (Figure 6), corresponds to the benzyl alcohol sitting flat on the electrode, with the benzyl ring essentially pi-stacking with the graphene surface (panel a, Figure 7). This is a clear example of the hydrophobic effect for the water-based electrolyte when the electrode is uncharged. It is essential to realize, however, that solvophobic forces are general and arise for solvents other than water, as clearly observed for the methanol electrolyte (Figure 6b). The next minimum at ∼5.3 Å corresponds to a structural motif in which the benzyl ring is tilted off the electrode surface, while the hydroxyl group is touching the electrode surface within the inner region of the EDL (shown in Figure 7, panel b). While this minimum is less stable than the closest minima at low-to-moderate surface charge, it becomes the primary surface association motif at high surface charge; when the EDL is highly concentrated, the substrate hydroxyl group can favorably interact with perchlorate anions and hydrogen bond to densely packed solvent molecules at the electrode surface. The last minimum is broad and shallow, located at approximately ∼7.5 Å from the electrode, and corresponds to the *p*-methoxybenzyl alcohol substrate away from the electrode surface, just beyond the dense ion/solvent layer formed at the electrode interface.

**Figure 7:**
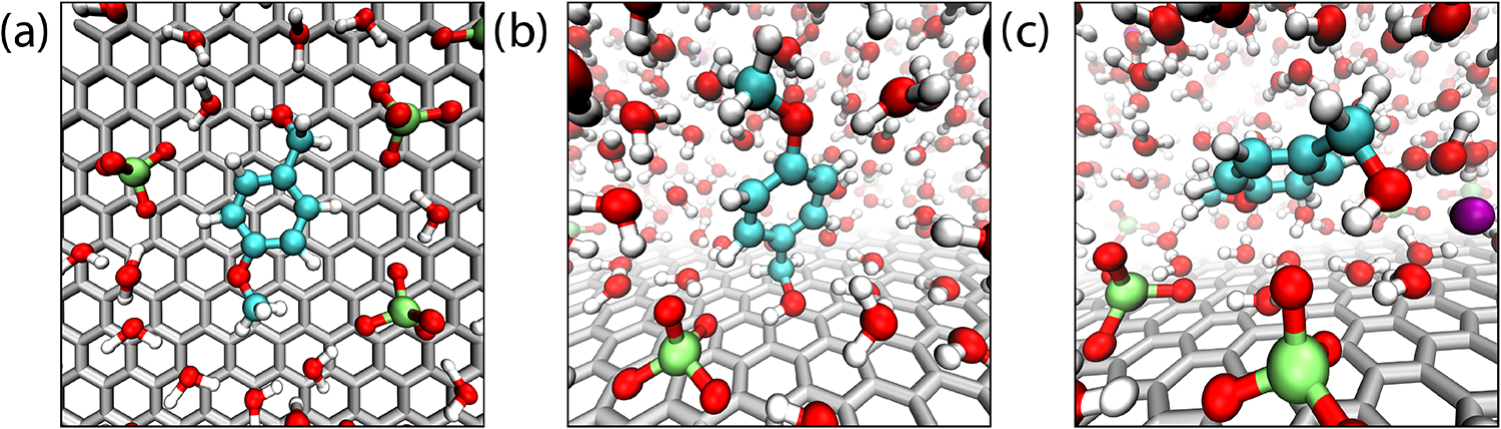
Snapshots of adsorption motifs of *p*-methoxybenzyl alcohol within an anodic EDL near the working electrode within 0.4 M LiClO_4_ aqueous electrolyte. Motifs depicted in a–c correspond to PMF minima at ∼3.5, 5.3, and 7.5 Å, respectively, that are observed in Figures 6 and 8. Reproduced with permission from ref 151. Copyright 2025 American Chemical Society.

The PMFs in Figure 6 have been computed for the neutral *p*-methoxybenzyl alcohol substrate before the initial electron transfer occurs. These PMFs are thus useful for generally investigating relevant solvophobic forces and the ability of the substrate to approach the electrode for initial electron transfer but do not provide information about the residence time of the cation radical intermediate (“B”, Figure 5) near the electrode, which is a key factor determining whether the electrosynthesis proceeds via the E-C-E or DISP mechanism. This information is instead provided by PMFs of the oxidized *p*-methoxybenzyl alcohol intermediate (i.e., cation radical) near the working electrode. PMFs for the corresponding cation radical intermediate are shown in Figure 8, as computed analogously to those for the neutral substrate. Lennard-Jones parameters were taken to be the same as for the neutral substrate (i.e., OPLS-AA force field^144^), with charges parametrized specifically for the cation radical oxidation state. Electrostatic interactions between the oxidized benzyl alcohol intermediate and both the positive electrode surface and anions within the anodic double layer modulate the PMFs shown in Figure 8, as a function of the electrode surface charge. For the oxidized intermediate, the motif of the benzyl ring “sitting flat” on the electrode (panel a, Figure 7) is only a local free energy minimum at low surface charge, while at moderate-to-high electrode charge, this configuration becomes highly unfavorable due to electrostatic repulsion between the electrode and the positive charge on the benzyl ring. The green curve(s) in Figure 8 show the PMF corresponding to the charge at working potential (σ ∼ 14.6 μC/cm^2^) for which the primary free energy minimum corresponds to the structural motif in (panel b), Figure 7, with the benzyl ring tilted off the electrode with the hydroxyl group touching the electrode surface. This favorable free energy minimum arises in part due to solvophobic forces but also because of electrostatics. The positively charged benzyl ring is positioned away from the positive electrode surface (to minimize repulsion) and situated approximately within the concentrated anionic double layer (to maximize attraction). Furthermore, the hydroxyl group remains touching the electrode surface to hydrogen bond with solvent molecules (and anions) concentrated near the electrode.

**Figure 8:**
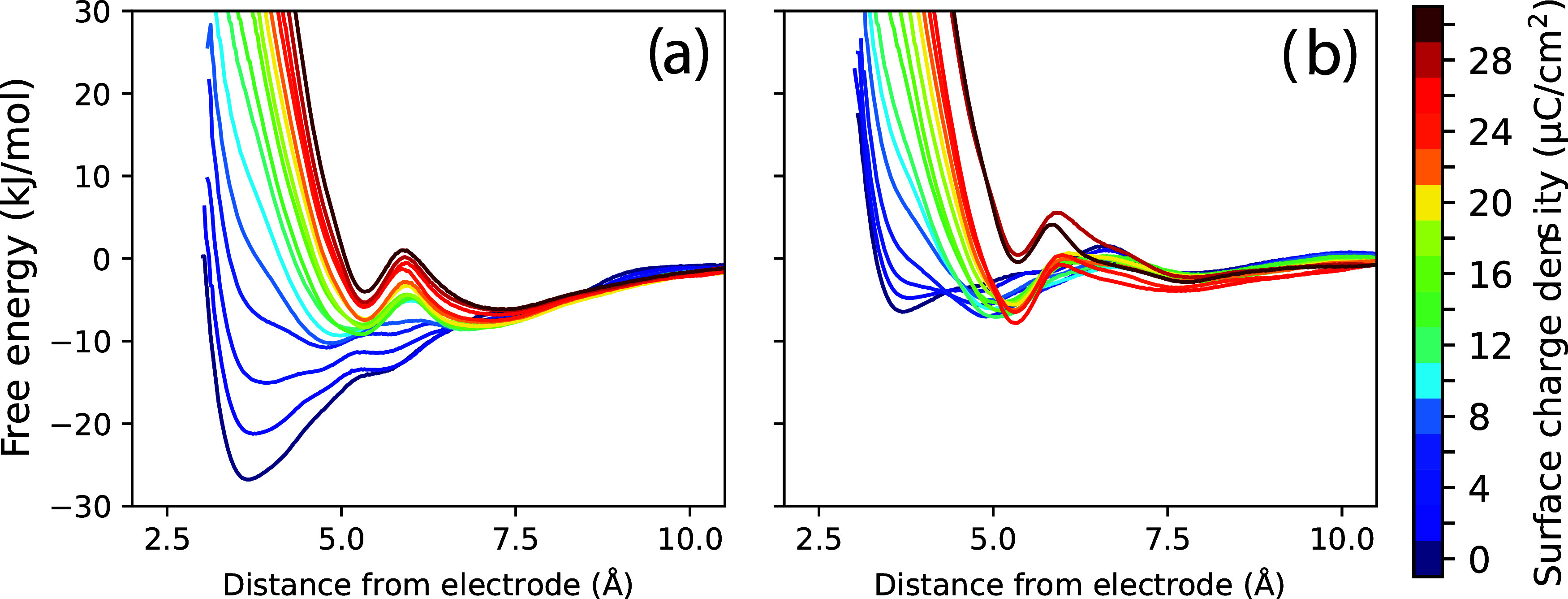
Free energy profiles (PMFs) for oxidized *p*-methoxybenzyl alcohol (i.e., cation radical intermediate) near the working carbon anode as a function of surface charge. (a) Water solvent with 0.4 M LiClO_4_ electrolyte; (b) methanol solvent with 0.4 M LiClO_4_ electrolyte. (a) is adapted with permission from ref 151. Copyright 2025 American Chemical Society.

An important takeaway from the PMFs in Figure 8 is that electrogenerated cation radicals can exhibit favorable association free energies with positively charged anode surfaces, at working potentials. This is due to both solvophobic forces and electrostatics. An organic cation radical substrate can typically rearrange/reorient to position its positive charge away from the positive electrode surface and centered within the concentrated anionic double layer, to optimize electrostatic interactions; this is consistent with our finding for other substrates.^124^ The result is that at working potential, the oxidized *p*-methoxybenzyl alcohol intermediate has an association free energy of −5 to −10 kJ/mol to the anode surface (motif in panel b, Figure 7), for both water- and methanol-based electrolytes (green curve(s), Figure 8). It is clear that the type of solvent/electrolyte can significantly alter the stability of the electrogenerated cation radical within the EDL, as the PMF dependence on electrode working charge differs markedly for the water and methanol electrolytes. In the methanol solvent, the shape of the PMF profile changes with surface charge (Figure 8b), due to a charge-induced reorientation of methanol molecules at the anode surface that influences the association of the cation radical with the electrode.

Quantitative values for the desorption rate constants “*k*_D_”, “*k*_D_^′^” corresponding to traversing the free energy barrier(s) in the PMFs (Figures 6 and 8) can then be computed from direct (unbiased) molecular dynamics simulations. Detailed methodology for this has been reported in the literature.^159^ Briefly, the substrate distance from the electrode is tracked over long MD trajectories to determine the average residence time τ within a given basin (e.g., free energy minimum) before moving beyond a predefined boundary. The inverse of this residence time provides the first-order rate constant, *k*_D_. The substrate is initially placed near the electrode surface and desorbs and readsorbs multiple times from the electrode during a long MD simulation; an upper wall potential is placed 20 Å from the electrode surface to prevent the substrate from diffusing too far into the bulk. The boundary for desorption from the electrode (and out of the inner double layer) is chosen as 15 Å from the electrode surface, at which the PMF profiles are flat (Figures 6 and 8). The average residence time τ for the benzyl alcohol substrate/intermediate near the electrode was computed from ten independent MD simulations of 250 ns each, totaling 2.5 μs of simulation time (for σ = 0 μC/cm^2^ charge, independent simulations were extended to 500 ns to sample the rare desorption events). In general, this direct MD approach can be used to predict rate constants with values of *k*_D_ ≥ 10^6^ s^–1^; this corresponds to average electrode residence times τ ≤ 1 μs, which would require batches of ∼10s μs long MD simulations for sufficient statistical sampling.

Rate constants “*k*_D_^′^”,“*k*_D_” for desorption of the neutral and oxidized *p*-methoxybenzyl alcohol substrate from the working electrode surface are given in Figure 9 (henceforth we generally refer to these rate constants as “*k*_D_”, although it should be understood they are different for the substrate/intermediate). As defined above, *k*_D_ = τ^–1^, where τ corresponds to the average time for the substrate/intermediate to desorb out of the free energy minima at the electrode surface (PMFs, Figures 6 and 8), to a distance of 15 Å away from the electrode surface (where the PMFs are flat). Figure 9 gives values for *k*_D_ computed for a large range of charge 0 ≤ σ ≤ 28 μC/cm^2^ on the working electrode, with working potential estimated as σ ∼ 14 μC/cm^2^. We first discuss “*k*_D_” values computed for the water-based electrolytes. When the electrode is uncharged (σ = 0 μC/cm^2^), the rate constant *k*_D_ ∼ 2–3·10^6^ s^–1^ is essentially the same for both the neutral and oxidized substrate (within prediction uncertainty). When the electrode is held at the working potential, *k*_D_ increases in magnitude as the free energy minima in the PMFs are shallower. For the neutral substrate *k*_D_ ∼ 2·10^7^ s^–1^, while for the oxidized intermediate *k*_D_ ∼ 1·10^8^ s^–1^ at the working potential (σ = 14 μC/cm^2^). The same trend continues as the electrode surface charge is further increased to σ = 28 μC/cm^2^, and the rate constant increases to values of *k*_D_ ∼ 2 to 3·10^8^ s^–1^ for both the neutral and oxidized substrate.

**Figure 9:**
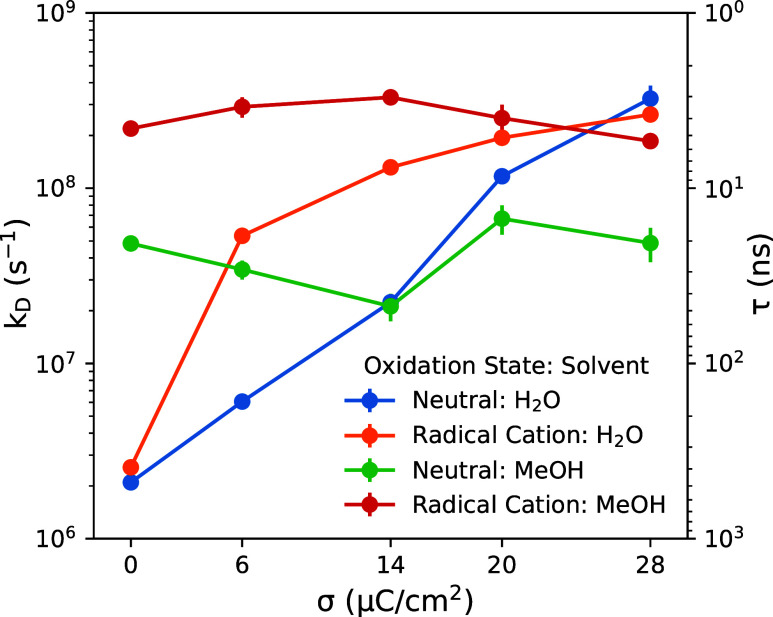
Rate constants “*k*_D_” and corresponding time constants τ (opposite *y*-axis) for desorption of *p*-methoxybenzyl alcohol from the working carbon electrode for the substrate in both neutral and oxidized states as a function of working electrode charge σ. Rate constants are computed for both water and methanol solvents with a 0.4 M LiClO_4_ electrolyte. Rate constant predictions for the aqueous electrolyte are taken from ref 151.

Corresponding rate constants “*k*_D_” within the methanol-based electrolytes are also given in Figure 9. The major difference is that for the methanol-based electrolytes, the rate constants “*k*_D_” are much less sensitive to electrode charge than for the aqueous electrolytes. For the neutral *p*-methoxybenzyl alcohol substrate, *k*_D_ ∼ 2 to 7·10^7^ s^–1^ and for the oxidized substrate, *k*_D_ ∼ 2 to 3·10^8^ s^–1^ over the full range of electrode surface charge 0 ≤ σ ≤ 28 μC/cm^2^; for comparison, rate constants vary by nearly 2 orders of magnitude for the aqueous electrolytes, over this electrode charge range. The explanation for this is that the free energy minima of the PMFs within aqueous electrolytes exhibit larger magnitude modulations with electrode surface charge compared to the PMFs within methanol electrolytes (Figures 6 and 8). Within the methanol-based electrolytes, both the rate constants “*k*_D_” and PMFs exhibit nonmonotonic dependence on electrode surface charge, which is due to the substantial structural changes in the inner layer of the EDL, involving methanol solvent reorientation with increasing electrode charge.

The quantitative values for “*k*_D_” are an essential factor governing whether the benzyl alcohol, electrosynthesis reaction in Figure 1 proceeds via the heterogeneous E-C-E or DISP mechanisms (Figure 5). We had previously argued that for fast electrical/chemical steps with (pseudo) first-order rate constants of order *k*_ET,2_^het^, *k*_C_ ∼ 10^9^ s^–1^, then a desorption rate constant *k*_D_ < 10^9^ s^–1^ may lead to the heterogeneous E-C-E pathway. For the oxidized (cation radical) *p*-methoxybenzyl alcohol intermediate at the working electrode potential (σ = 14 μC/cm^2^), desorption rate constants are indeed below this threshold of *k*_D_ < 10^9^ s^–1^, and thus the heterogeneous E-C-E pathway would be likely given a fast chemical (deprotonation) step (*k*_C_ ∼ 10^9^ s^–1^, pseudo-first-order). Cation radicals are typically superacids,^160,161^ with p*K*_a_ ∼ −7 ± 2 for the *p*-methoxybenzyl alcohol cation radical (in acetonitrile),^162,163^ so one might a priori expect the rate constant *k*_*C*_ to be large for the C_α_–H deprotonation. However, pseudo-first-order rate constants for the C_α_–H deprotonation of *p*-methoxybenzyl alcohol (and related) cation radicals with water solvent have been experimentally measured as *k*_C_^hom^ ∼ 10^4^ s^–1^.^162^ Such slow deprotonation kinetics is anomalous relative to inorganic acids of comparable p*K*_a_ (with rate constants reaching the diffusion limit for the latter^164–166^ ), and is rationalized by an “intramolecular” electron transfer from the C_α_–H bond to the aromatic π system accompanying the deprotonation.^163,167^ The slow deprotonation step means that benzylic alcohol oxidation (Figure 1) may instead proceed via a DISP mechanism (and not E-C-E). In Section 4.2, we will show an example of how the heterogeneous and homogeneous proton transfer could have vastly different rate constants *k*_C_^het^ ≫ *k*_C_^hom^, for which the E-C-E mechanism would be much more likely.

The desorption rates “*k*_D_” for the substrates/intermediates to leave the electrode surface/inner double layer region will play an increasingly important role in the electrosynthesis mechanism (E-C-E vs DISP) for large, bulky substrates that are subject to strong solvophobic forces. In this regard, it is important to note that the rate constants “*k*_D_” are only partially (but not entirely) determined by the one-dimensional free energy profiles (PMFs) shown in Figures 6 and 8. For example, the desorption rate constants “*k*_D_” are order(s) of magnitude smaller than what would be predicted using a transition state theory estimate based on the free energy barriers from Figures 6 and 8. The reason is that the true transition state for the desorption process cannot be represented by just the substrate/electrode distance variable, but rather likely depends on one or more orthogonal “collective solvation coordinate(s)”. Due to the highly charged nature of the EDL, and charged substrate in the case of cation radical intermediate, there is substantial solvation “rearrangement” around the substrate and double layer ions as the substrate moves in/out of the inner double layer region. The desorption rate constant “*k*_D_” thus reflects the activation free energy associated with this solvation reorganization, and the true transition state is not adequately described in the one-dimensional free energy projections (PMFs) in Figures 6 and 8. This situation is entirely analogous (albeit more complex) to the process of ion pair dissociation in water, for which a solvation coordinate is essential for representing the dissociation transition state.^168^ Determining the true transition state and correspondingly the (many-body) solvation coordinates necessary to describe the substrate/electrode desorption process would require other approaches (e.g., transition path sampling^168^) and is beyond the scope of this article.

We conclude this Section by noting that the desorption rate constants “*k*_D_” and consequently the rate control of E-C-E vs DISP pathways (Figure 5) will vary considerably for different substrates, solvent and electrolyte types, and electrode working potentials. In Figure 10, we show computed adsorption free energies (PMFs) for a 1-naphthalenemethanol substrate, which can be compared to the PMFs for the *p*-methoxybenzyl alcohol substrate in Figure 6. The major difference is the bulkier “naphthyl” compared to the “benzyl” group, such that the 1-naphthalenemethanol substrate exhibits stronger solvophobic association with the carbon working electrode within the LiClO_4_/MeOH electrolyte, with larger magnitude minima in the PMFs (Figure 10 compared to Figure 6). Correspondingly, the desorption rate constants “*k*_D_” are substantially smaller in magnitude for the 1-naphthalenemethanol substrate compared to those of the *p*-methoxybenzyl alcohol substrate. Within the LiClO_4_/MeOH electrolyte for the electrode near working potential (σ = 11 μC/cm^2^), we compute a desorption rate constant of *k*_D_ ∼ 2·10^6^ s^–1^ for the 1-naphthalenemethanol substrate as compared to the value of *k*_D_ ∼ 1–2·10^7^ s^–1^ for the *p*-methoxybenzyl alcohol substrate at similar working conditions (Figure 9). Additionally, in Figure 10, we show PMFs computed at identical electrode working charge (either σ = 0, 11, or 23 μC/cm^2^), but with different bulk electrolyte concentration ranging from 0.1 to 0.5 M. The conclusion is that the PMFs are much more sensitive to working electrode charge compared to bulk electrolyte concentration, because it is the former that primarily determines the ionic concentration within the EDL (at least for moderate concentrations and large working potentials). The net charge in the double layer must neutralize the working electrode charge, and the double layer thus is typically much more concentrated than the bulk. For example, the ClO_4_^–^ anion concentration in the anodic double layer is 2.7–3.1 M at working electrode charge σ = 23 μC/cm^2^ for both 0.1 and 0.3 M bulk electrolyte concentrations; in comparison, the EDL anion concentration is 2.2 M for the 0.5 M bulk electrolyte system when the working electrode charge is lower, σ = 11 μC/cm^2^.

**Figure 10:**
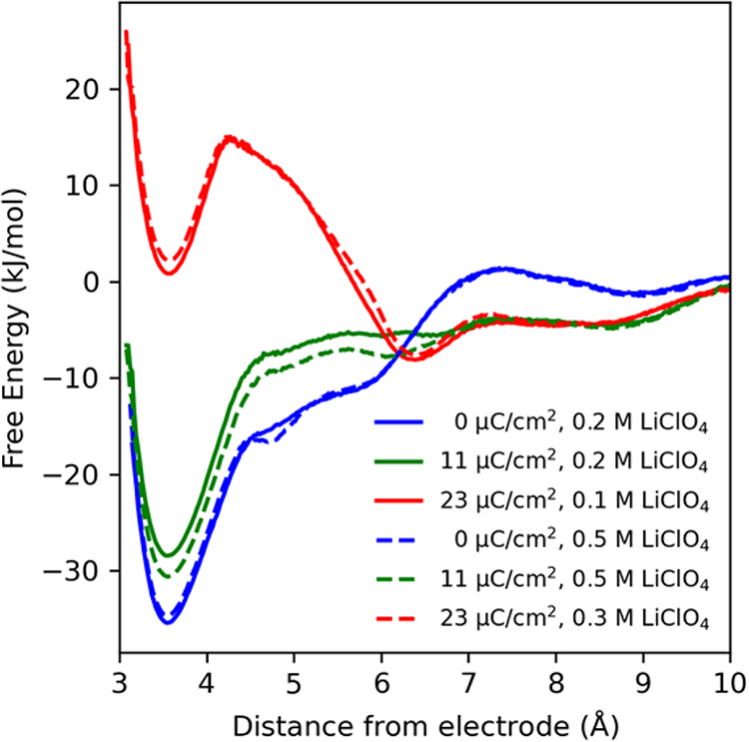
Free energy profiles (PMFs) for 1-naphthalenemethanol near a working carbon anode as a function of surface charge and bulk ion concentration within the LiClO_4_/MeOH electrolyte.

## 4 Electrical Double Layer Modulation of Cation Radical Reaction Kinetics

In this section, we discuss computation of rate constants *k*_C_^het^ for chemical reactions of electrogenerated intermediates. In all cases considered here, the reactive intermediate is a cation radical formed from initial substrate oxidation at the anode. We assume that transition state theory can be applied so that the prediction of *k*_C_^het^ is effectively equivalent to the computation of activation free energies of the elementary reaction step. The chemical rate constant *k*_C_^het^, along with the desorption rate constant *k*_D_ discussed in the previous section (as well as the electron transfer rate *k*_ET,2_^het^), will determine the branching between heterogeneous E-C-E and homogeneous DISP pathways. Besides distinguishing between E-C-E and DISP pathways, computation of *k*_C_^het^ provides detailed insight into how the EDL environment modulates the reaction kinetics of the cation radical intermediates, which may be beneficial for further optimizing reaction conditions (solvent, electrolyte, etc.) to maximize desired product yield and selectivity.

Our restricted focus on direct electrolysis at an inert electrode (without catalytic role of electrode surface) means that hybrid quantum mechanics/molecular mechanics (QM/MM) simulations^157,169–174^ are well suited for computing reaction free energies of the electrogenerated intermediates with atomistic resolution of the electrical double layer (EDL) environment. We note that much of the mechanistic insight presented in the case studies below requires explicit atomistic description of the EDL, and would not be derived from implicit or continuum solvent models. While alternative, ab initio molecular dynamics (AIMD) approaches, e.g., DFT description of full system, would be advantageous for case studies in which the electrode is catalytic, AIMD entails substantially greater computational cost compared to QM/MM, and would be intractable for the system sizes and free energy sampling discussed here. For the case studies below, the QM region consists of only the electrogenerated cation radical intermediate and certain solvent/electrolyte molecules involved in the reaction, while the electrode is modeled classically at the molecular mechanics level. A force field description of the electrode mitigates problems associated with artificial charge delocalization between redox couples/electrodes that can arise from self-interaction error within DFT functionals.^175–177^ It is also the case that empirical force fields are often more accurate than DFT for modeling the liquid properties of solvents/electrolytes.^178,179^

In the following case studies, reaction free energies were computed by utilizing DFT-based QM/MM molecular dynamics simulations, in tandem with free energy sampling techniques. Simulations are performed utilizing the PyDFT-QMMM software,^180^ which utilizes the Psi4 ^181^ and OpenMM^150^ software packages as QM and MM engines/libraries, respectively. Within the QM/MM simulation, forces are computed from a Kohn–Sham Hamiltonian with added MM and QM/MM coupling terms; the QM/MM coupling involves standard electrostatic embedding plus mechanical embedding for Lennard–Jones interactions.^180^ The PyDFT-QMMM software enables electrostatic embedding treated with a standard cutoff/truncation (typically chosen as, e.g., ∼1.4 nm) with whole molecules being embedded “on-the-fly” based on whether their centroid falls within the cutoff radius. Alternatively, PyDFT-QMMM enables a numerically exact, particle mesh Ewald-based electrostatic embedding protocol (termed “QM/MM/PME”),^182^ which is particularly well suited for capturing long-range electrostatics for QM/MM simulations of electrochemical cells. The PLUMED2 software package^183^ is interfaced as a plug-in to PyDFT-QMMM, to enable the myriad biased sampling/free energy sampling algorithms that are necessary for computing reaction free energies. In the case studies below, the employed free energy sampling techniques range from umbrella sampling or metadynamics^184–186^ using standard collective variables (CVs), to more difficult cases of deprotonation reactions that require more specialized/careful consideration of CVs and biased sampling procedures.^187–189^ We refer the interested reader to reference 180 discussing the PyDFT-QMMM implementation and reference 124 discussing a detailed computational electrosynthesis case study for more details.

We briefly comment on the computational cost of the DFT-QM/MM free energy simulations discussed below. Typical QM region sizes are ∼10–30 heavy atoms, modeled with standard choices of hybrid density functionals (e.g., PBE0, B3LYP, with D3 corrections) and double-ζ basis sets with polarization functions (e.g., 6–31G*, def2-SVP). The computational cost of each DFT-QM/MM MD step is almost entirely determined by the cost of the underlying self-consistent field (SCF) and the corresponding analytical gradient evaluation. Within Psi4, the SCF/gradient evaluations typically take 10–60 s wall time for such QM systems, as run on 8 CPU cores. For the smaller QM systems, this means that a DFT-QM/MM MD trajectory of tens of picoseconds can be generated in several days of wall time. Each of these simulations will correspond to “one umbrella potential”, within umbrella sampling generation of the free energy profile, or alternatively, tens of simulations are run in parallel within multiwalker metadynamics^184–186^ to perform the free energy sampling. Finally, we should mention that DFT exhibits well-known errors for describing the electronic structure of cation radicals (due to self-interaction error),^190–192^ and it is wise to a priori benchmark levels of theory (functional/basis set) to higher level calculations (e.g., MP2 gas-phase or implicit solvent) for the substrate of interest. In our experience, we have found that good results can be achieved by exploiting error cancellation with appropriate functional/basis set choice,^193^ but because such error cancellation is not robust, it should be determined on a system-specific basis.

### 4.1 Intramolecular Coupling of Cation Radical Intermediates from Electron-Rich Olefins: Influence of EDL Electrostatics and Electrode Surface Interactions

The first case studies that we discuss are based on anodic intramolecular coupling reactions that have been experimentally conducted by Moeller and co-workers.^6,30,194,195^ The relevant substrates typically contain enol ether or dithioketene acetal initiator groups that exhibit lower oxidation potentials compared to unfunctionalized olefins, and are oxidized to cation radical intermediates (via outer-sphere electron transfer) at anodic potentials of ∼1–1.5 V vs Ag/AgCl.^196,197^ Compared to bare olefins, these electrogenerated cation radical species are relatively protected against deprotonation pathways, and strategic incorporation of substrate nucleophilic groups can be utilized as the synthetic route to a variety of cyclization products via oxidative formation of C–C, C–O, or C–N bonds.^6,14,16,30,194–198^

In Figure 11, we show a schematic for an anodic intramolecular coupling reaction of 1-methoxy-1,6-octadiene substrate to form a six-membered cyclization product. The initial mechanistic steps have been experimentally determined to proceed via an E-C-E mechanism, in which the chemical “C” step corresponds to intramolecular nucleophilic attack of the enol ether cation radical by the secondary olefin group on the substrate. This cyclization step is likely reversible for the cation radical intermediate, and the cyclization product is “locked-in” only after the second oxidation step; in this regard, the rate of the second electron transfer step has been deemed important for successful product formation.^194^ The second electron transfer gives a dication intermediate, and solvent methanol trapping, followed by deprotonation, leads to the final product. Experimentally, direct electrolysis of the 1-methoxy-1,6-octadiene substrate in methanol-based electrolytes leads to both 5-membered and 6-membered ring products in moderate yield, as previously reported by Moeller and co-workers.^195^

**Figure 11:**
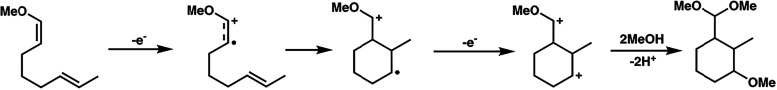
Schematic of E-C-E-based reaction mechanism for anodic intramolecular cyclization of the 1-methoxy-1,6-octadiene substrate. Diagram shows a 6-membered ring cyclization product, but a 5-membered ring product is also possible. Adapted with permission from ref 193. Copyright 2024 American Chemical Society.

There are several reasons to a priori postulate that anodic intramolecular coupling reactions typical to those conducted by the Moeller group,^6,30,194,195^ proceed via heterogeneous E-C-E rather than the DISP pathway. First, the electrolysis (Figure 11) is often done in a methanol-based solvent, and it has been empirically demonstrated that alcohols effectively trap enol ether-based cation radicals.^11,14,198^ Thus, the intramolecular coupling “C” step (Figure 11) must be fast to outcompete side reactions involving methanol/solvent trapping of the cation radical. Given a large chemical rate constant for the intramolecular coupling *k*_C_, the reaction will proceed via heterogeneous E-C-E if the desorption rate constant for the cation radical intermediate *k*_D_ < *k*_C_, *k*_ET,2_^het^ (Figure 5), with *k*_ET,2_^het^ expected to be large given the normally significant overpotential for the second electron transfer.^124^ For the large, bulky organic substrates that are often targets for anodic coupling reactions,^14,16,30,197,198^ slower rates of substrate/intermediate desorption from the electrode surface (i.e., *k*_D_) are expected due to strong solvophobic forces, as discussed in Section 3. For a variety of substrates, Moeller and co-workers have shown unequivocally that such anodic coupling reactions proceed via the heterogeneous E-C-E pathway, based on findings that the product yield/selectivity depends sensitively on the rate of the second (heterogeneous) electron transfer.^30,194^

How then might the EDL environment modulate the kinetics of the chemical step (*k*_C_^het^) for the radical cation intermediate, and thus potentially alter the product yield/selectivity for the electrolysis (Figure 11)? Indeed, Moeller and co-workers have previously shown that tuning the EDL composition substantially improves product yield for related anodic, intramolecular coupling reactions.^14^ To investigate this, DFT-QM/MM molecular dynamics simulations were utilized to compute reaction free energies for the cation radical intermediates, within the EDL environment under working conditions. Here, we focus on the reaction free energy for the target intramolecular cyclization step involving the cation radical intermediate, although it is also useful/important to investigate possible side reactions as well (i.e., methanol/solvent trapping).^193^ Figure 12a shows the free energy surface for cyclization of the 1-methoxy-1,6-octadiene cation radical intermediate to either five-membered or six-membered ring conformers, within the EDL of a 0.5 M Li^+^/ClO_4_^–^/MeOH electrolyte at carbon (graphite) anode at working potential. The free energy surface is plotted in terms of the two carbon–carbon distances R_C_2_/C_6__ and R_C_2_/C_7__ that were biased with umbrella potentials in the free energy sampling, as chosen to distinguish between 5-membered and 6-membered ring conformers. There are different possible stereoisomer products, and (Figure 12a) specifically considers the “SS” 6-membered ring and “SR” 5-membered ring products, as shown in the Figure inset. The reaction free energy was computed for the most probable substrate location within the EDL, corresponding to a free energy minimum in computed electrode distance PMFs (i.e., similar to Figure 8). A more detailed description of the computational methods and analysis is given in reference 124.

**Figure 12:**
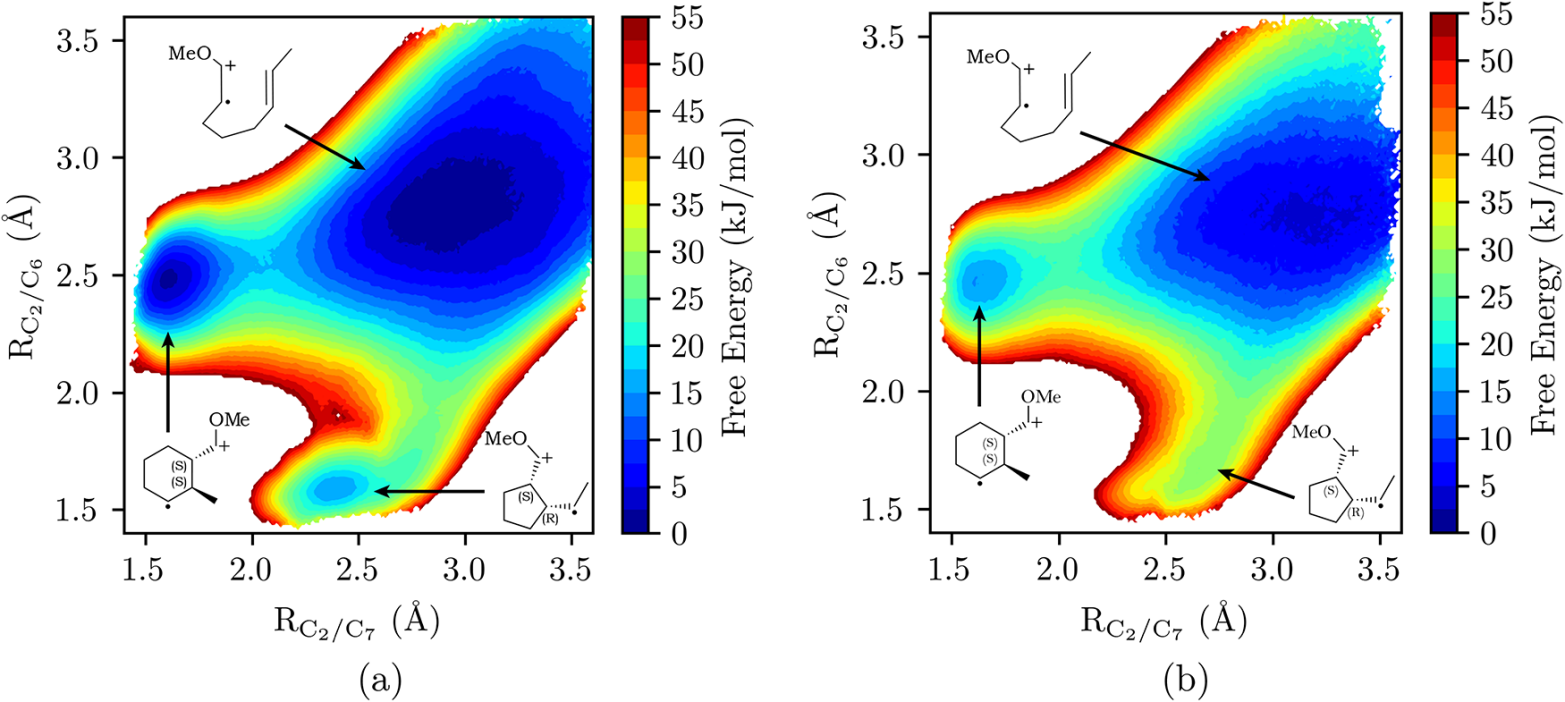
Cyclization reaction free energy profiles for the 1-methoxy-1,6-octadiene cation radical intermediate. (a) Reaction occurring within the EDL of 0.5 M Li^+^/ClO_4_^–^/MeOH electrolyte at carbon (graphite) anode at working potential and (b) reaction within the bulk methanol solvent. Adapted with permission from ref 124. Copyright 2023 American Chemical Society.

To directly illustrate the EDL modulation of the reaction free energy in (Figure 12a), the analogous reaction free energy surface was computed in bulk methanol solvent for reference, as shown in (Figure 12b). By comparing (Figure 12a and Figure 12b), it is evident that the EDL environment significantly modulates the cation radical reaction free energy, promoting greater stabilization of the cyclic cation radical 5-membered and 6-membered ring conformers. The two cyclized conformers correspond to the localized free energy minima (blue regions) on the left and bottom regions of the free energy profile, as indicated by the molecular structures shown in the inset; the broad free energy minimum on the top-right region of the profile corresponds to the uncyclized cation radical structure. The EDL environment promotes stabilization of the 6-membered conformer by ∼15–20 kJ/mol, and stabilization of the 5-membered conformer by ∼5–10 kJ/mol (as relative to the uncyclized conformer), compared to the bulk methanol environment. The specific electrostatic interactions of the cation radical intermediate with anions within the EDL that lead to the modulated reaction free energy are discussed thoroughly in reference 124. The positive charge is more localized (on the C_α_–H group) for the cyclic structures (5-membered/6-membered ring) compared to the uncyclized cation radical intermediate, leading to enhanced stabilization of the cyclic intermediates via Coulombic attraction with double-layer anions. Electrostatic repulsion between the cation radical intermediate and positive working electrode is minimized due to the specific structural arrangement of the substrate on the working electrode surface.^124^

EDL modulation of the cation radical reaction shown in Figure 12 is an example of “electrostatic catalysis”,^33–36,38,43,46^ in which strong electric fields and ion–ion interactions near the working electrode may substantially alter activation barriers and/or the stabilization of short-lived intermediates (e.g., cyclic structures, Figure 12) that are key to the overall electrochemical reaction yield and selectivity. Besides the much enhanced ion concentrations in the double layer, ionic interactions with electrogenerated radical ion intermediates are particularly strong due to low dielectric screening within the EDL, with solvent molecules exhibiting an effective dielectric constant dramatically reduced compared to the bulk (e.g., ϵ ∼ 5–10 for water in the Stern layer).^39–42^ The EDL effect on the cation radical cyclization reaction may explain, in part, empirical observations that yield/selectivity depend on electrochemical reaction conditions (e.g., solvent, electrolyte, etc.).^6,30,194,195^ Indeed it was previously found that the EDL free energy modulation was much more significant for the 0.5 M Li^+^/ClO_4_^–^/MeOH electrolyte as shown in Figure 12, compared to the analogous reaction occurring within an EDL of a 0.5 M triethylmethylammonium/tetrafluoroborate (TEMA^+^/BF_4_^–^) electrolyte in acetonitrile solvent.^124^ This is because, in the latter electrolyte, there is substantial ion pairing between the BF_4_^–^ anions and TEMA^+^ cations within the anodic double layer, leading to fewer “free anions” to interact with the cation radical intermediate.

For reactions occurring at a heterogeneous electrode–electrolyte interface, in addition to electrostatic and solvation effects discussed above, there are additional steric effects that may modulate the activation free energy. If the electrogenerated intermediate reacts while in contact with the electrode surface, the electrode surface imparts steric constraint on the configurations/rearrangement of the substrate during the reaction. Such heterogeneous, steric effects on reactivity have been demonstrated empirically, specifically for substrates with aryl rings that “stack” against the electrode surface.^52^ As an example, we have computationally investigated steric effects on a representative anodic coupling reaction to form cyclic amines, as studied experimentally by the Moeller group.^30^ The mechanistic steps of this reaction are shown in Figure 13. Initial oxidation of the dithioketene acetal group at the anode gives the electrogenerated cation radical, which can undergo nucleophilic attack by the imine group to form a five-membered heterocyclic intermediate. A second oxidation followed by methanol trapping and subsequent deprotonation leads to the neutral methoxylated intermediate, which leads to the cyclic amine product upon an acid–based, hydrolysis workup. Because the substituted imine group of the initial substrate can be generated via condensation of an amine with benzaldehyde or related derivatives, this approach enables the possibilities for (1) orthogonal selectivity/reactivity compared to amine-based cyclizations, and (2) introduction of an auxiliary group (–R group, Figure 13) to potentially influence the reaction.^30^

**Figure 13:**
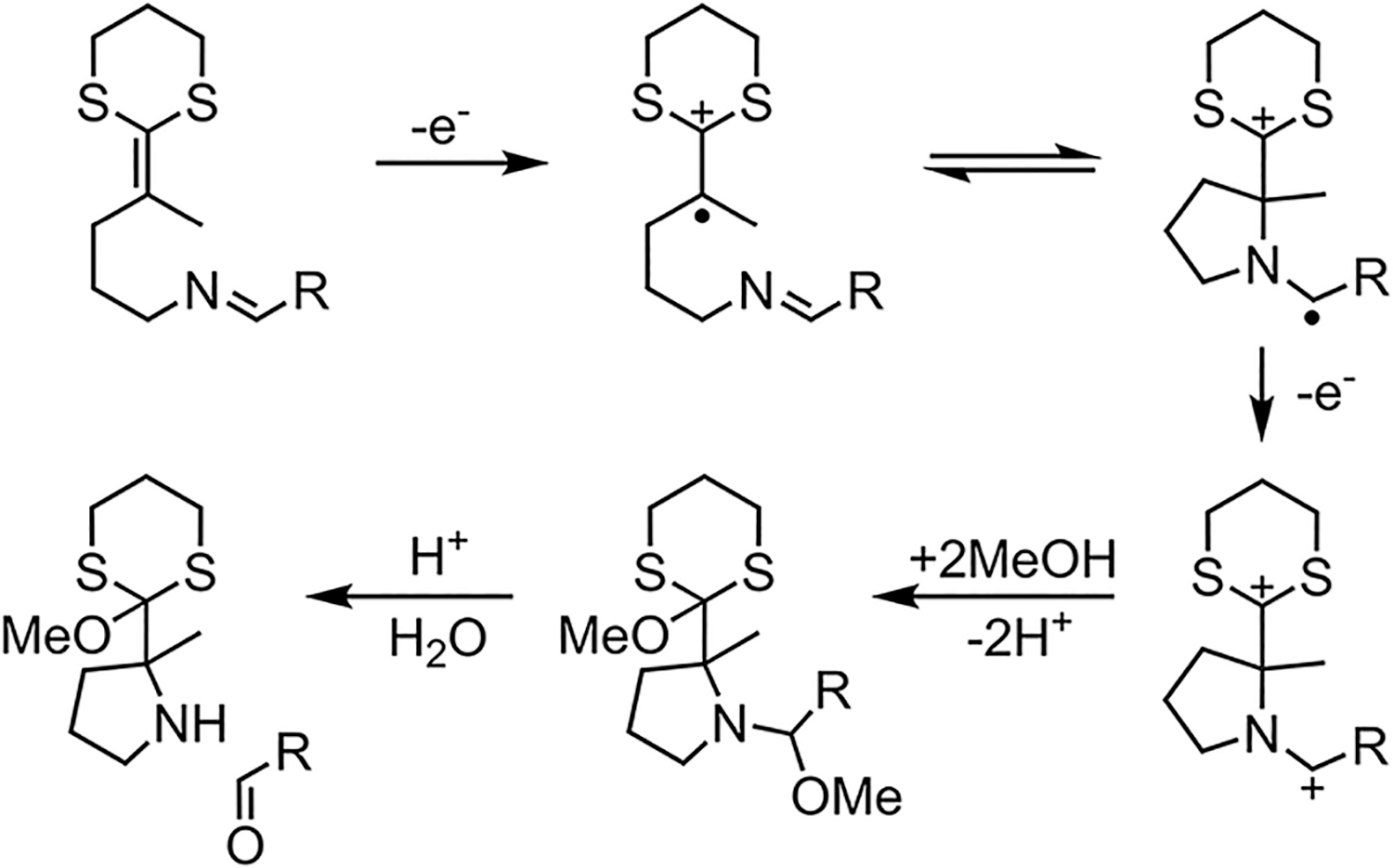
Schematic of the E-C-E-based reaction mechanism for anodic intramolecular cyclization of dithioketene acetal substrate with an imine nucleophile.

There are two key aspects to note about such anodic cyclization reactions of the dithioketene acetal substrates, shown in Figure 13. Anodic intramolecular coupling enables the formation of a 5-membered ring product, whereas a typical acid-catalyzed cyclization between an electron-rich olefin and imine would lead to a 6-membered ring product formed via an iminium cation intermediate. The 6-membered ring product is not observed in the electrochemical process because it would require a difficult oxidation of an N-centered radical intermediate.^30^ Another important note is that such imine-based cyclizations require dithioketene acetal rather than enol ether initiators due to the lower oxidation potential of the former. The requirement is a lower oxidation potential of the initiator group compared to the formed cyclic amine product, which is only achieved by using the more electron-rich olefin initiator (i.e., dithioketene acetal).^30^

The Moeller group has previously shown that the anodic coupling reactions shown in Figure 13 are compatible with a variety of benzylic, naphthyl, or biphenyl auxiliary groups.^30^ We focused on the naphthyl auxiliary group (R = naphthyl, Figure 13), as it was anticipated that this substrate could exhibit particularly strong interactions with the electrode due to both large solvophobic forces and steric effects. Similar to our previous computational study,^124^ we focused on the cyclization step of the cation radical intermediate, and utilized DFT-based QM/MM molecular dynamics with free energy sampling to compute the cyclization reaction free energy profile. An electrochemical cell was constructed with a carbon working electrode (anode), with an applied surface charge of σ ∼ 8.5 μC/cm^2^, approximately corresponding to the working potential as estimated by the procedure described in Section 2. The electrolyte studied consisted of a 50:50 methanol/THF solvent mixture, with 0.1 M tetraethylammonium/tosylate (Et_4_NOTs) ions, as motivated by the experimental electrolysis conditions.^30^ Computed PMFs (not shown) indicate that the naphthyl-functionalized substrate exhibits a substantial association free energy of ∼−25 kJ/mol with the anode surface at applied working potential, in large part due to the strong solvophobic association of the naphthyl group with the carbon electrode. While we did not explicitly compute residence time for the substrate/intermediate at the working electrode surface, we estimate that this residence time may be hundreds of nanoseconds or more, based on the results/discussion for similar substrates presented in Section 3.

DFT-QM/MM free energy simulations at the PBE0-D3/6–31G level of theory were conducted to compute the cyclization free energy profile of the cation radical intermediate shown in Figure 13 (with R = naphthyl). We computed the reaction free energy within the anodic electrochemical double layer, with the substrate residing in the free energy minimum, as associated with the electrode surface. For comparison, we also computed the reaction free energy within bulk methanol solvent, as a baseline from which to interpret the double-layer/steric effects on the free energy profile. The computed free energy profiles are shown in Figure 14. Two distance coordinates (C–C and C–N) between the imine C/N atoms and carbon coupling site were used as variables for 2D umbrella sampling simulations, with a total of 225 umbrella windows and 12 ps of QM/MM MD sampling at each umbrella. Figure 14a shows a simulation snapshot of the substrate at the free energy minima associated with the working electrode surface. It is clearly observed that the naphthyl group is situated flat against the electrode surface, with the dithioketene acetal, cation radical group oriented away from the electrode surface and residing within the electrical double layer.

**Figure 14:**
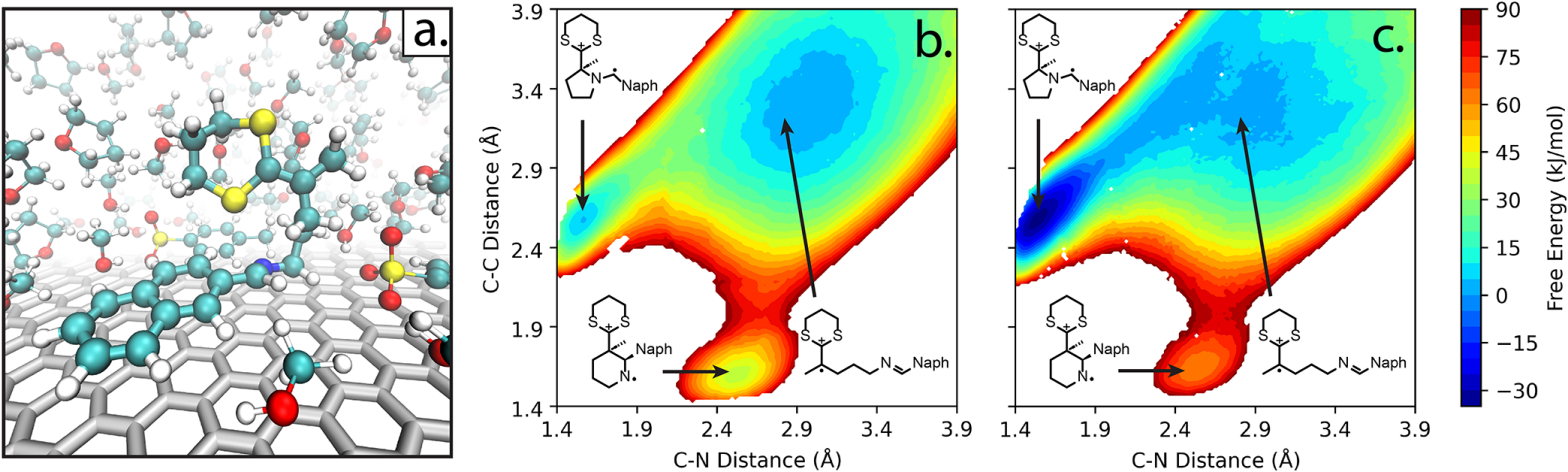
Cyclization reaction free energy profiles for dithioketene acetal, cation radical intermediate. (b) reaction occurring within the EDL of 0.1 M Et_4_NOTs electrolyte in a 50:50 methanol/THF solvent mixture; (c) reaction within the bulk methanol solvent. (a) Snapshot from the QM/MM simulation of the cation radical substrate with the naphthyl group stacked against the anode surface.

The QM/MM computed free energy profiles in Figure 14 show a clear difference in the (cation radical) cyclization reaction free energy for the reaction occurring at the anode surface compared to the reaction occurring in bulk solvent (methanol). In bulk methanol solvent, cyclization to the 5-membered ring via imine trapping of the dithioketene cation radical is thermodynamically favorable and occurs with barrierless kinetics. In this case, the thermodynamic driving force is ∼30–50 kJ/mol (depending on how the “reactant geometry” is defined), indicating that the imine group is a very effective nucleophile for trapping the dithioketene cation radical. This computational finding is consistent with empirical observations by Moeller and co-workers that the yield of such reactions is not significantly affected when LiOMe base is added to the electrolyte;^30^ imine nucleophilic trapping of the cation radical can apparently outcompete trapping by methoxide, as consistent with the fast reactions suggested by our computed free energy profiles (Figure 14). Note that the QM/MM free energy profiles also predict a local minimum corresponding to the 6-membered cyclic cation radical structure (Figure 14), but this secondary minimum is much higher in free energy, with a substantial kinetic barrier, and a corresponding 6-membered cyclic product is not observed experimentally. As mentioned, the 6-membered cyclic product formation is likely prohibited due to a subsequent step involving the difficult oxidation of the N-centered radical.^30^

Comparison of Figure 14b with Figure 14c indicates that while the cyclization reaction is still quite facile when it occurs at the anode surface, it is both thermodynamically and kinetically hindered compared to the corresponding reaction in the bulk solvent environment. At the electrode, the cyclized and uncyclized cation radical geometries exhibit nearly the same free energy, and there is a kinetic barrier of ∼25 kJ/mol for the cyclization step occurring at the electrode. The simulation snapshot in Figure 14a indicates that the primary reason for this altered reaction kinetics is likely the steric effects imposed by the stacking of the naphthyl group against the carbon electrode surface. As mentioned, the substrate exhibits strong solvophobic attraction (∼25 kJ/mol) to the electrode (at working potential) largely due to this naphthyl/electrode stacking, which positions the nucleophilic imine group also stacked against the electrode surface (Figure 14a). In contrast, the dithioketene cation radical group is positioned within the double layer, away from the electrode surface. Nucleophilic coupling would thus require movement of the cation radical electrophile from the EDL toward the electrode surface, or movement of the imine/naphthyl groups off the electrode and into the EDL toward the electrophile; both processes would incur free energy cost and thus explain the observed kinetic barrier when the reaction occurs at the electrode surface (Figure 14b). We note that because this steric effect largely originates from solvophobic forces mediating the stacking of the naphthyl group and carbon electrode, the explicit solvent description enabled by the QM/MM simulations is likely crucial for modeling this process (i.e., would not be captured with implicit solvent models).

The overall takeaway is that the intramolecular coupling reaction shown in Figure 14 is likely to occur via the E-C-E pathway (vs DISP) due to the relatively fast kinetics of intramolecular nucleophilic attack of the imine group to the dithioketene cation radical. This conclusion is consistent with the empirical observation by Moeller and co-workers that the yield/selectivity of this class of reactions is modulated by the rate of the second heterogeneous electron transfer step.^30^ For the cation radical cyclization step, a moderate (∼25 kJ/mol) kinetic barrier may arise due to steric effects of, e.g., the naphthyl auxiliary group stacked against the carbon electrode, but the mediating solvophobic forces will also lead to an increased residence time (hundreds of nanoseconds) at the working electrode, giving the cation radical more time to cyclize and subsequently undergo the second heterogeneous electron transfer. The free energy calculations depicted in Figures 12 and 14 for the two different anodic coupling reactions, indicate that the working electrode and EDL environment can modulate the reaction kinetics of electrogenerated intermediates in various ways. DFT-QM/MM free energy calculations thus serve as a powerful tool to elucidate such electrostatic and steric effects on reaction kinetics, which may play an important role in dictating the overall reaction mechanism (E-C-E vs DISP) and thus the observed yield and selectivity of the electrosynthesis process.

### 4.2 Deprotonation of Benzylic Alcohol Cation Radical Intermediate: Comparison of Heterogeneous vs Homogeneous Reaction Kinetics

We now resume the discussion of the anodic electrolysis of benzyl alcohol substrates to their aldehydes, with the reaction scheme depicted in Figure 1. In Section 3, we presented a detailed analysis of rate constants *k*_D_ for *p*-methoxybenzyl alcohol (and its oxidized intermediate) to desorb from the carbon anode held at working potential, within an aqueous LiClO_4_ electrolyte. In this section, we discuss the computations of the chemical rate constant *k*_C_, which corresponds to the deprotonation of the cation radical intermediate following initial oxidation. Kinetic competition between this deprotonation step occurring heterogeneously at the anode surface (*k*_C_^het^) and the desorption rate constant of the cation radical intermediate (*k*_D_) likely dictates whether the electrolysis reaction proceeds heterogeneously via E-C-E or homogeneously via the DISP pathway. This is because the second electron transfer is expected to be fast if it occurs heterogeneously at the anode. Indeed, previous DFT/implicit solvent calculations of the first and second oxidation potentials corresponding to the reaction in Figure 1, predict that the second oxidation potential is nearly ∼2 eV lower than the first oxidation potential.^151^ This means that heterogeneous electron transfer for the second oxidation would proceed via a very large rate constant *k*_ET,2_^het^ = *k*_ET,2_^het,0^ e^(1−α)*F*(*E*–*E* ^0^)/*RT*^, given an overpotential of nearly *E* – *E* ^0^ ∼ 2 V when the anode is held at a working potential corresponding to the first oxidation process.

As discussed in Section 3, despite the *p*-methoxybenzyl alcohol cation radical being a superacid with a very low p*K*_a_ (p*K*_a_ ∼−7 ± 2 as measured in acetonitrile^162,163^ ), deprotonation rate constants of this cation radical have been experimentally measured as *k*_C_^hom^ ∼ 10^4^ s^–1^ (pseudo-first-order) in water solvent,^162^ indicating a moderate deprotonation barrier of ∼0.4–0.5 eV. Indeed, Savéant and co-workers have found that such barriers are typical for deprotonation of aromatic cation radicals, and are rationalized by the C_α_–H bond dissociation initially proceeding via a homolytic cleavage (H^•^), before switching to a heterolytic cleavage (H^+^) only after “intramolecular electron transfer” to the aromatic π system.^167^ A similar rationale for experimentally measured activation barriers of alkylaromatic cation radical deprotonation reactions has been given in terms of stereoelectronic arguments.^163,199,200^ To facilitate deprotonation, one of the electrons in the C_α_–H σ bond must be transferred to the aromatic pi system of the benzyl ring, which is facilitated if there is conjugation/overlap between the orbital on the C_α_–H carbon atom with the pi-framework of the benzyl ring, requiring a specific molecular configuration.^163,200^ The takeaway/consequence of this (relatively) slow deprotonation step is that anodic electrolysis of benzylic alcohols is likely to proceed via a DISP mechanism given the magnitudes of the desorption rate constants *k*_D_ discussed in Section 3, unless the deprotonation barrier is reduced (vide infra).

In previous work,^151^ DFT-QM/MM simulations were utilized to compute reaction free energies for the deprotonation of the *p*-methoxybenzyl alcohol cation radical intermediate. Electrolysis conditions with two different aqueous electrolytes were investigated, with either 0.4 M LiClO_4_ or 0.4 M NaOAc salt. In the LiClO_4_ aqueous electrolyte, water molecule(s) serve as the base, so deprotonation of the cation radical produces a solvated hydronium (H_3_O^+^) ion in addition to the benzyl radical (Figure 1). In the NaOAc electrolyte, acetate serves as the base to produce acetic acid as an intermediate. Both heterogeneous and homogeneous deprotonation reactions were investigated, in which deprotonation occurs within the anodic EDL in the former and within the bulk water solution in the latter. To investigate the heterogeneous reaction in the double layer, PMFs were first computed to find the most probable/favorable location of the cation radical intermediate near the working anode surface.^151^ Distinct minima were observed in the PMFs, and the QM/MM reaction free energy computations were conducted at one of these chosen minima. Figure 15a shows a snapshot of the *p*-methoxybenzyl alcohol cation radical corresponding to one of the PMF minima near the working anode; as is visually evident, the substrate is situated with its alcohol group touching the working electrode surface.^151^

**Figure 15:**
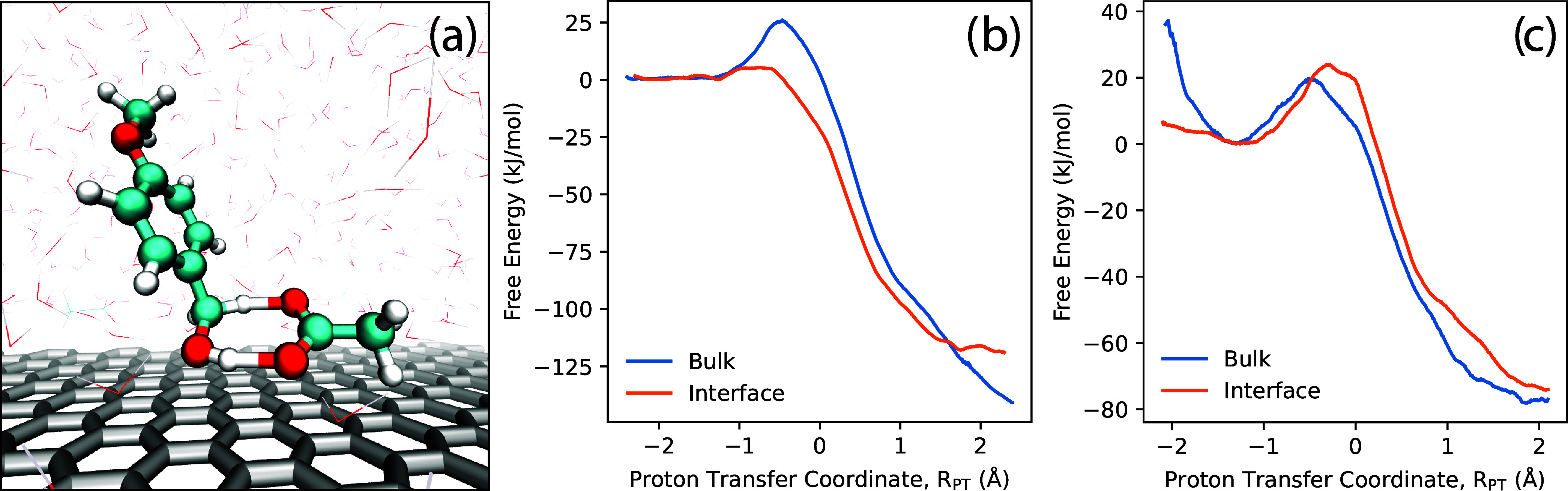
Free energy profiles for deprotonation of *p*-methoxybenzyl alcohol cation radical; (b) deprotonation to acetate base in NaOAc aqueous electrolyte; (c) deprotonation to water base in LiClO_4_ aqueous electrolyte. Free energy profiles are shown for reactions within bulk water (blue curves) and within the anodic double layer at the electrode surface (“Interface” orange curves). (a) Snapshot of the transition state for *p*-methoxybenzyl alcohol cation radical deprotonation to acetate base at the anodic electrochemical interface. Adapted with permission from ref 151. Copyright 2025 American Chemical Society.

Figure 15 shows free energy profiles for deprotonation of the *p*-methoxybenzyl alcohol cation radical to the acetate base in NaOAc aqueous electrolyte (Figure 15b) and the water base in LiClO_4_ aqueous electrolyte (Figure 15c). In both cases, reaction free energies were computed within bulk water (“Bulk” blue curves) and within the anodic double layer at the electrode surface (“Interface” orange curves). As mentioned, “Interface” deprotonation reactions correspond to QM/MM free energy simulations of the *p*-methoxybenzyl alcohol cation radical situated within a minima of the PMF for the substrate to reside near the working anode. In all cases deprotonation is very thermodynamically favorable (as expected given the cation radical p*K*_a_ ∼ −7 ± 2 ^162,163^ ), and the focus is the activation free energy barrier that dictates the reaction kinetics. For deprotonation to water base in LiClO_4_ aqueous electrolyte (Figure 15c), the activation barrier is computed as ∼20–25 kJ/mol regardless of whether the reaction proceeds homogeneously in bulk water (blue curve) or heterogeneously at the anode interface (orange curve). This computed barrier is lower than the experimentally measured value of ∼0.4–0.5 eV,^162^ and this discrepancy was attributed (to some extent) to the level of DFT theory (functional/basis set) utilized in the QM/MM free energy predictions.^151^ While DFT exhibits well-known errors/deficiencies for describing reactions of organic radical ions,^190–192^ relative comparisons of QM/MM predictions as to the effect of the solvation environment are expected to be qualitatively robust.

Turning to deprotonation of the *p*-methoxybenzyl alcohol cation radical to acetate base in NaOAc aqueous electrolyte (Figure 15b), the activation barrier is computed as ∼25 kJ/mol within the bulk solution (blue curve). Thus, in the bulk solution, deprotonation to either water or acetate base exhibits a similar activation barrier, despite the large shift in thermodynamics with the stronger acetate base. This is in line with previous experimental findings that deprotonation rate constants for the PMBA cation radical do not necessarily correlate with the strength of the associated base.^163^ However, a major unexpected finding was the dramatic reduction in activation barrier for the deprotonation reaction to acetate base occurring at the anode interface; the computed barrier drops to only ∼5 kJ/mol for the heterogeneous interfacial reaction in aqueous NaOAc electrolyte (orange curve, Figure 15b) compared to the value of ∼25 kJ/mol within bulk water solution.

Figure 15a shows a snapshot from the QM/MM simulation of the transition state for deprotonation of the *p*-methoxybenzyl alcohol cation radical to acetate base occurring at the anode interface. The acetate anion forms a hydrogen bond with the cation radical alcohol group situated on the electrode surface, simultaneously with the other oxygen atom abstracting the proton from the acidic C_α_–H group. This “bidentate” character of the acetate ion is seemingly an important factor in the reduced deprotonation barrier. The reduction in activation barrier was demonstrated to arise from stereoelectronic effects.^151^ As mentioned, prior experiments have concluded that deprotonation of alkylaromatic cation radicals requires conjugation/overlap of the carbon orbital with the pi-framework of the benzyl ring to facilitate intramolecular electron transfer;^163,200^ such orbital alignment occurs if the C_α_–H bond is oriented perpendicular to the plane of the benzyl ring (as is observed in the Figure 15a snapshot). It was found that the anodic double layer environment biases the configurations of the cation radical/acetate complex toward such orientations, thus reducing the stereoelectronic contribution to the activation barrier.^151^ This is thus a compelling example of how the electrostatic, steric, and solvophobic forces imposed by the electrochemical interface can alter the stereoelectronics of the reaction complex, corresponding to a substantial change in the reaction kinetics (as computationally predicted).

The reduction in the activation barrier for cation radical deprotonation to acetate is not the full story with regard to the influence of the anodic double layer on the reaction kinetics. The reaction occurs via second-order kinetics, and with the bulk acetate concentration being 0.4 M in the electrolyte, one might estimate (based on concentrations) kinetically slow diffusive formation of the reactive “encounter pair” complex. In this regard, another effect of the double layer is to bias the preformation of the cation radical/acetate “encounter pair” complex. First, the concentration of acetate anions is enhanced by more than an order of magnitude within the anodic double layer compared to the bulk, driven by the positive charge on the anode.^151^ Additionally, the favorable orientation of the *p*-methoxybenzyl alcohol cation radical with its alcohol group situated on the anode surface (Figure 15a) promotes complexation with acetate via the strong acetate/alcohol hydrogen bond. Simulations verify that the cation radical/acetate “encounter pair” complex is highly likely to be “pre-formed” in the double layer, and thus eliminates any diffusive contribution to the second-order rate constant. The overarching conclusion is that the cation radical deprotonation kinetics is likely significantly faster within the NaOAc aqueous electrolyte. There may be substantial practical consequences for yield/selectivity of the benzylic alcohol electrolysis reaction if this computational prediction is valid. Rapid deprotonation would favor the heterogeneous E-C-E pathway instead of the homogeneous DISP pathway, with an expected difference in benzyl radical intermediate lifetime given differences in rates (homogeneous vs heterogeneous) for the second oxidation/electron transfer. The benzyl radical intermediate is susceptible to numerous side reactions, including hydrogen atom abstraction or dimerization, and in this case, coupling with benzyl aldehyde product to a 1,2-diol, and thus the lifetime of this intermediate determines the susceptibility to side reactions. The computational prediction of dramatically modulated heterogeneous reaction kinetics (Figure 15), is thus an example of how mechanistic insight derived via computation may be used to tune electrosynthesis reaction conditions to optimize product yield/selectivity.

### 4.3 Role of the Solvent in Intramolecular Alcohol Trapping of Enol Ether Cation Radical

Because electrogenerated organic cation radicals are often highly acidic (as discussed above), the solvation environment may play an important role in stabilizing acidic protons of reaction intermediates. The benzylic alcohol oxidation example discussed above indicates how the specific solvation environment may modulate the deprotonation rate constant for a key step in the electrochemical reaction pathway. Nucleophilic coupling reactions are another common/prototypical reaction pathway of electrogenerated cation radicals, for which the solvent may play a key role in stabilizing an acidic proton(s) during the reaction step(s). For example, alcohol and amine nucleophiles will exhibit acidic protons following coupling to a cation radical electrophile, and the solvent may play an important role in stabilizing such acidic protons during and after the coupling step.

As an example, we consider an anodic intramolecular coupling reaction similar to the examples discussed in Section 4.1, but with an alcohol group acting as the nucleophile in the cyclization step with the enol ether cation radical. The specific reaction is shown in Figure 16. In this reaction, a tetramethoxyhexenol substrate undergoes direct electrolysis to produce a tetramethoxyfuranose derivative product, as experimentally demonstrated by Moeller and co-workers.^14^ Following initial oxidation, the cyclization step involves intramolecular nucleophilic attack of the alcohol group to the enol ether cation radical, followed by deprotonation/second oxidation and methanol trapping to the final product (Figure 16). It has been experimentally shown that the cation radical cyclization step is reversible, and the rate of the second electron transfer is an important factor dictating the observed product yield.^16,201^ Furthermore, it has been empirically found that the reaction yield is highly variable to the composition of the reaction media, with yield/selectivity depending on the nature of the solvent (e.g., methanol vs THF) and the type of electrolyte (e.g., LiClO_4_ vs Et_4_NOTs).^14^

**Figure 16:**
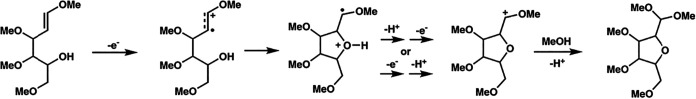
Schematic of reaction mechanism for anodic intramolecular cyclization of a tetramethoxyhexenol substrate. Adapted with permission from ref 193. Copyright 2024 American Chemical Society.

To investigate solvent effects on the reaction in Figure 16, DFT-QM/MM free energy simulations were previously conducted, specifically focusing on the chemical step involving cyclization of the cation radical.^193^ While the full electrochemical reaction likely occurs heterogeneously at the anode surface (e.g., E-C-E),^16^ for simplicity, the reaction was computationally studied in bulk methanol solvent, as sufficient for elucidating the specific role of methanol in the coupling reaction. Full computational details may be found in reference 193. The free energy profile for the cation radical cyclization reaction is shown in Figure 17a. The 2D free energy profile was generated via umbrella sampling along reaction coordinates corresponding to the distance between the −OH nucleophile (oxygen atom) and each of the two carbon atoms of the enol ether cation radical group. The uncyclized cation radical substrate corresponds to the top-right region of the free energy profile, where there is no corresponding free energy minima, while the two free energy minima observed correspond to the 5-membered heterocycle (at R­(C_β_–*O*_*H*_) ∼ 1.5 Å) and 6-membered heterocycle (at R­(C_α_–*O*_*H*_) ∼ 1.5 Å).

**Figure 17:**
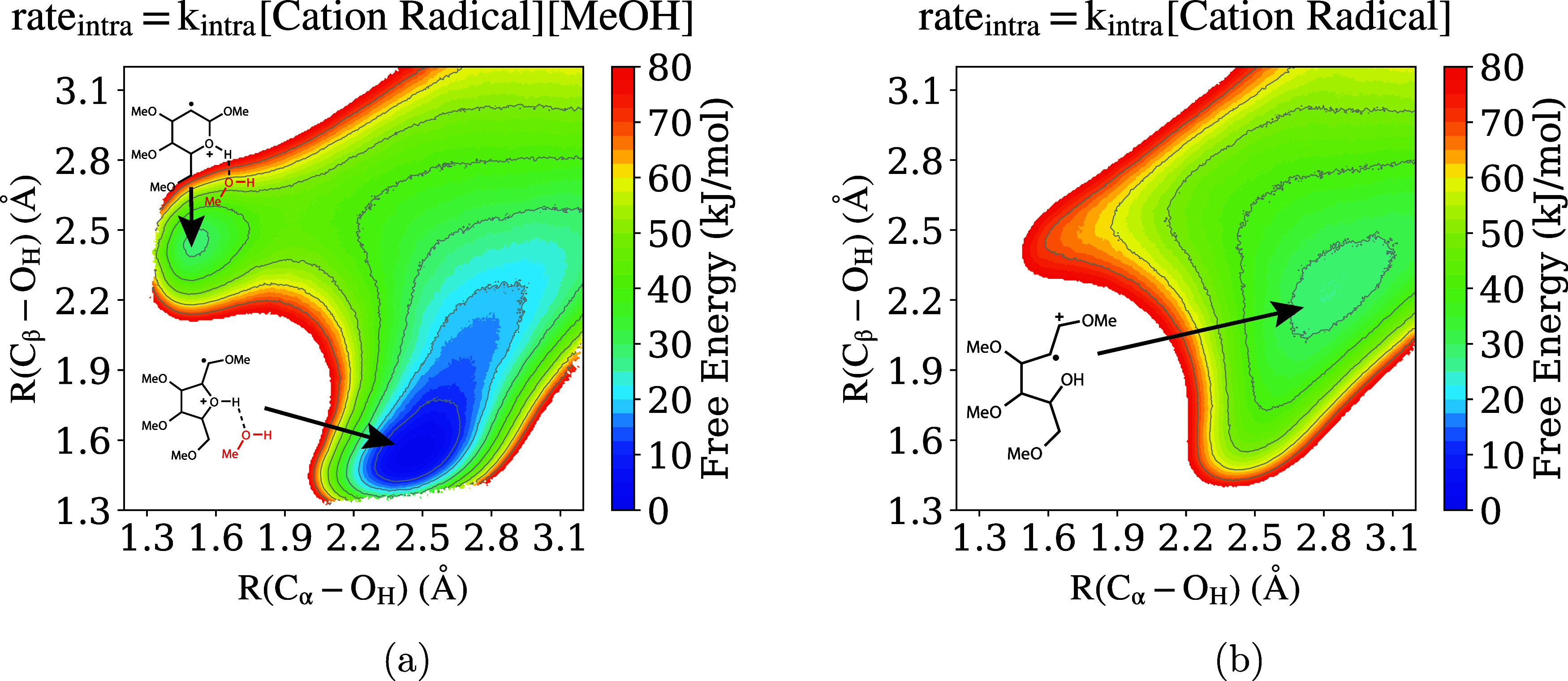
Free energy profile for cyclization of tetramethoxyhexenol cation radical, involving intramolecular coupling of alcohol nucleophile to the oxidized enol ether group, as computed within bulk methanol solvent. The reaction coordinates R­(C_α_–*O*_*H*_) and R­(C_β_–*O*_*H*_) correspond to distances between the −OH nucleophile (oxygen atom) and each of the two carbon atoms of the enol ether cation radical group. (a) Free energy surface computed by including a methanol solvent molecule and the substrate in the “QM” region of the QM/MM Hamiltonian, and (b) the corresponding profile with just the substrate (and no methanol) in the “QM” region. Reproduced with permission from ref 193. Copyright 2024 American Chemical Society.

The 5-membered heterocycle corresponds to the experimentally observed product,^14^ and the computed free energy profile indicates that the cation radical cyclization step (in Figure 17a) is thermodynamically favorable and with barrierless kinetics. This cyclization step should thus occur rapidly at the anode surface, facilitating heterogeneous electron transfer for the second required oxidation step (Figure 16). An essential factor facilitating the cyclization is the complexation of methanol solvent with the acidic −OH proton of the cyclic cation radical intermediate. Before cyclization, the −OH proton is only mildly acidic, but it becomes strongly acidic following nucleophilic attack on the enol ether cation radical. Upon cyclization, a methanol solvent molecule complexes to this acidic proton, forming a shared-proton or “Zundel-like” structure, with short O–H distances of ∼1.1 and ∼1.3 Å between the acidic proton and oxygen atoms of the methanol and substrate. This solvent complexation provides a substantial contribution to the free energy driving the cation radical cyclization process. To demonstrate the solvent contribution, in Figure 17b we show a corresponding DFT-QM/MM free energy profile but computed in the absence of methanol solvent complexation. To be explicit, in Figure 17a, the “QM” region included both the cation radical substrate and complexing methanol solvent molecule (allowing for “chemical” interactions between methanol/substrate), while in Figure 17b only the cation radical substrate was included in the “QM” region and all methanol solvent molecules were treated as “MM” (thus incorporating “electrostatic” but not “chemical” methanol/substrate interactions). The dramatic difference in the free energy profiles of Figure 17a and Figure 17b (and lack of stable cyclized structures in the latter) clearly indicates the substantial influence of methanol complexation for driving the cation radical cyclization (via stabilization of the acidic proton).

There is an important corollary/conundrum to the observation that the alcohol trapping reaction illustrated in Figure 17a is kinetically barrierless. Methanol-based solvents/electrolytes are commonly utilized for anodic electrosynthesis processes for reactions that proceed via electrogenerated cation radicals;^202^ in this case, wouldn't methanol/solvent attack of the cation radical intermediate lead to detrimental side reactions that might outcompete the intended reaction pathway? ^14,16^ To investigate this, additional QM/MM free energy simulations were conducted (not shown here) to explore methanol trapping side reactions of the cation radical substrate; indeed, it was found that methanol trapping was also a kinetically facile/low barrier reaction.^193^ Thus, while methanol is necessary to facilitate the reaction, being important to “catalyze” the cation radical cyclization step (Figure 17a), and is involved in the last chemical step following deprotonation and second oxidation (cation trapping to form acetal, Figure 16), the methanol solvent introduces kinetically competing side reactions that may affect product yield. There are two important points to note about this kinetic competition. First, there is an important rate order difference: the intramolecular cyclization proceeds via overall second-order kinetics (Figure 17a), being first order in both substrate and methanol concentration. Methanol solvent enters the rate equation due to the required driving force contribution from methanol complexation (i.e., Figure 17a vs Figure 17b). In contrast, solvent/methanol intermolecular trapping of the enol ether cation radical proceeds via overall third-order kinetics, being second order in methanol concentration. The reason for this rate order dependence is similar; when methanol attacks the cation radical, the methanol proton becomes highly acidic, requiring a second methanol molecule to complex and form a shared-proton configuration.^193^ The other important aspect to the kinetic competition between intra- and intermolecular alcohol trapping is that particular substrate conformations may disfavor the intermolecular trapping due to both steric and electronic factors,^193^ but such effects will clearly be substrate-specific.

Within the electrical double layer, such solvent effects on cation radical reaction kinetics may be increasingly pronounced due to both strongly modulated (local) concentration profiles and electrostatic/steric effects discussed in Section 4.1. At large working potentials, the EDL is highly concentrated in ions, with solvent molecules being highly structured/ordered at the electrode interface. There may be limited availability of “free solvent” molecules to participate in the third-order kinetics of the intermolecular trapping reaction, such that the kinetic competition between target and side reactions may be highly tunable/variable with electrolyte/solvent choice and composition (as observed empirically).^14^ Indeed, additional computational studies in our group indicate that the discussed paradigm is general to other types of cation radicals, other protic nucleophiles (amines in addition to alcohols), and other weakly basic solvents (THF in addition to methanol). While the computational predictions shown in Figure 17 clearly demonstrate the central solvent effect, DFT-QM/MM free energy simulations of the reaction within the full electrochemical system (similar to simulations presented in Section 4.1) would enable the direct characterization of analogous effects within the double layer environment.

### 4.4 Nucleophilic Attack vs Deprotonation Pathways of Olefin Cation Radicals within Ionic Liquid-Based EDLs

The several examples discussed in Sections 4.1 and 4.3 demonstrate how electrochemical oxidation of electron-rich olefins (e.g., enol ether or dithioketene acetal electroauxiliary groups^202^) is a strategic pathway to mediate intramolecular coupling reactions. What about the related oxidative functionalization of simple olefins via corresponding anodically generated cation radical intermediates? Given the ubiquity of olefin groups and thus possibility of synthetic utility, there is a long history of such reactions based on direct electrolysis of unfunctionalized olefins (i.e., without electroauxiliary group);^11,203,204^ however, often the yield/selectivity of the electrolysis is observed to be low. The uncatalyzed electrochemical process is typically hampered by several fundamental challenges. The first challenge is that unfunctionalized olefins exhibit large oxidation potentials of ∼2–3 V vs Ag/AgCl for outer-sphere electron transfer, which is beyond the electrochemical stability window of most standard solvents/electrolytes that are utilized in electrosynthesis.^11^ The second challenge is that even if the desired cation radical could be electrogenerated without oxidizing solvent/electrolyte, there is typically uncontrolled reactivity of this cation radical intermediate, given the fast kinetics for both deprotonation and solvent nucleophilic attack.

An example of such an anodic electrolysis pathway is shown in Figure 18 for the tetramethylethylene substrate. Following oxidation of tetramethylethylene (**1**), the cation radical **2** is susceptible to either nucleophilic attack or deprotonation by solvent molecules; methanol or acetic acid are common solvents utilized in such prior experimental work.^11,203^ Here we consider the case of a water-containing electrolyte in which water molecules act as either the nucleophile or base. Deprotonation of the allylic hydrogen of **2** leads to allylic radical intermediate **3** that can be further oxidized to form an allylic carbocation. A competing pathway is water nucleophilic attack on **2** to form the oxonium cation radical intermediate **4**, which then can undergo deprotonation and second oxidation to the 2,3-dimethyl-2-butanol cation. The final cation intermediates shown in Figure 18 will inevitably undergo subsequent reactions (e.g., nucleophilic attack), but we do not consider such subsequent steps here. Prior experimental work suggests that both pathways, involving deprotonation of **2** to **3** or water nucleophilic attack on **2** to form **4**, are kinetically competitive and the relative branching depends on nucleophilicity of the solvent.^203^ Clearly, kinetic control of one pathway versus the other would be desirable to facilitate a higher yield/selectivity electrosynthetic outcome.

**Figure 18:**
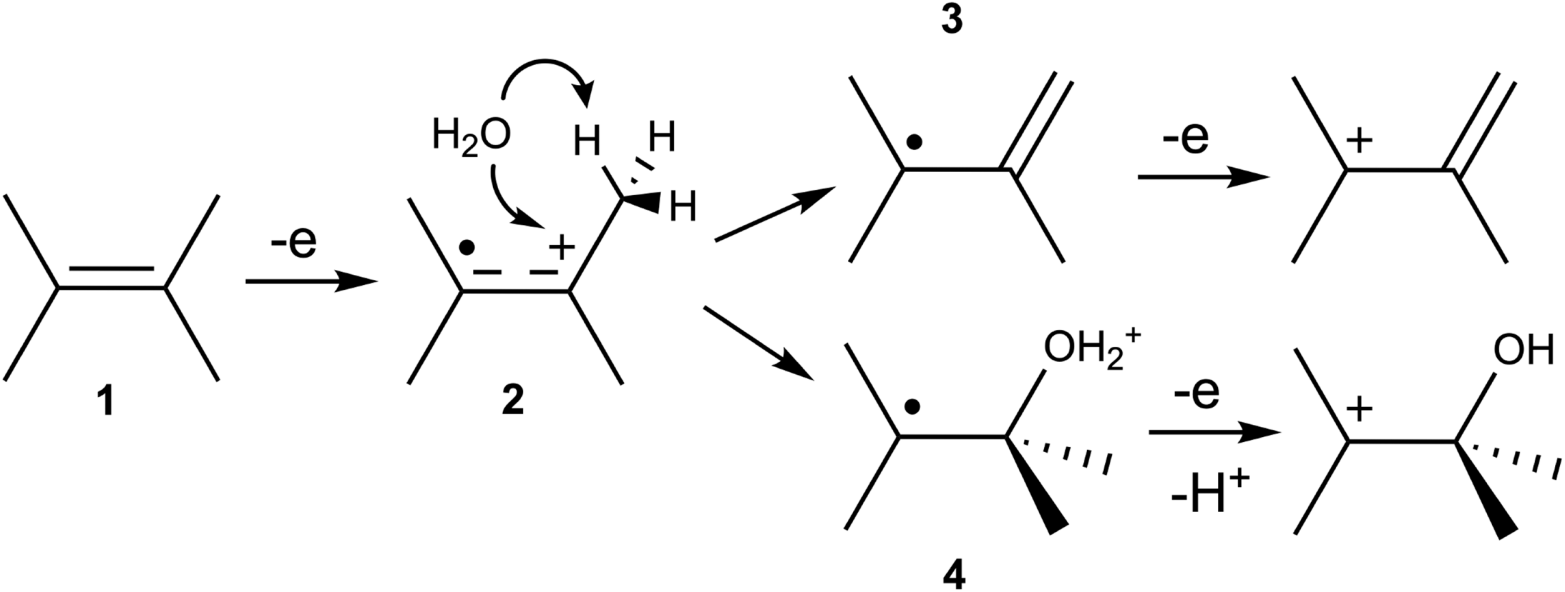
Schematic of reaction mechanism for anodic electrolysis of tetramethylethylene in a water-containing electrolyte. The electrogenerated cation radical **2** can undergo deprotonation by water to intermediate **3**, or direct nucleophilic attack by water to intermediate **4**. Following a second oxidation (and deprotonation for **4**), these pathways lead to cation intermediates that undergo further reaction.

We hypothesize that such kinetic control may be achievable via custom design or tuning of the electrolyte. We considered an ionic liquid electrolyte with dilute water content, focusing on the prototypical 1-butyl-3-methylimidazolium tetrafluoroborate (BMIM/BF_4_) ionic liquid as an example. Ionic liquids are advantageous in that they have large electrochemical stability windows;^10^ furthermore, dilute water in ionic liquids has widened electrochemical stability as well (due to reduced water activity), as has been demonstrated extensively in the analogous case of water-in-salt electrolytes.^51,205^ This makes the electrochemical stability of a low-water content, ionic liquid feasible at the necessary anodic potentials for such outer-sphere, olefin oxidation (e.g., ∼2 V vs Ag/AgCl). Furthermore, it has been well-demonstrated that the water microenvironment for dilute water in ionic liquids is highly tunable, via selection of various “hydrophobic” or “hydrophilic” ionic liquids.^206^ Because the stability of the hydronium (H_3_O^+^) cation (generated from deprotonation) and alkyloxonium cation **4** (generated from nucleophilic attack) will be highly sensitive to the local water microenvironment, tuning this microenvironment may enable substantial changes in the kinetic competition between the deprotonation and nucleophilic attack pathways shown in Figure 18.

As a preliminary investigation, we conducted QM/MM free energy simulations for the water deprotonation/nucleophilic attack of the tetramethylethylene cation radical (**2**) within BMIM/BF_4_ and different water microenvironments. The QM/MM Hamiltonian utilized B3LYP-D3/def2-SVP level of DFT theory in combination with previously developed for force fields for similar systems.^207,208^ QM/MM free energy simulations utilized multiwalker metadynamics^184–186^ with 36 walkers for 45 ps MD trajectories to bias sample the two collective variables corresponding to C–O bond formation from nucleophilic attack, and C–H bond breaking during deprotonation. Two different water microenvironments were considered; first, a three water molecule cluster (“3-water”) reacting with the tetramethylethylene cation radical, and second a five water molecule cluster (“5-water”) reacting with the tetramethylethylene cation radical; in both cases, the substrate/water clusters are solvated within BMIM/BF_4_ ionic liquid. Figure 19a shows a snapshot from the 5-water QM/MM simulation depicting the deprotonation transition state. In each simulation, the tetramethylethylene cation radical and water cluster comprised the “QM” region, while the surrounding BMIM/BF_4_ ionic liquid comprised the “MM” region.

**Figure 19:**
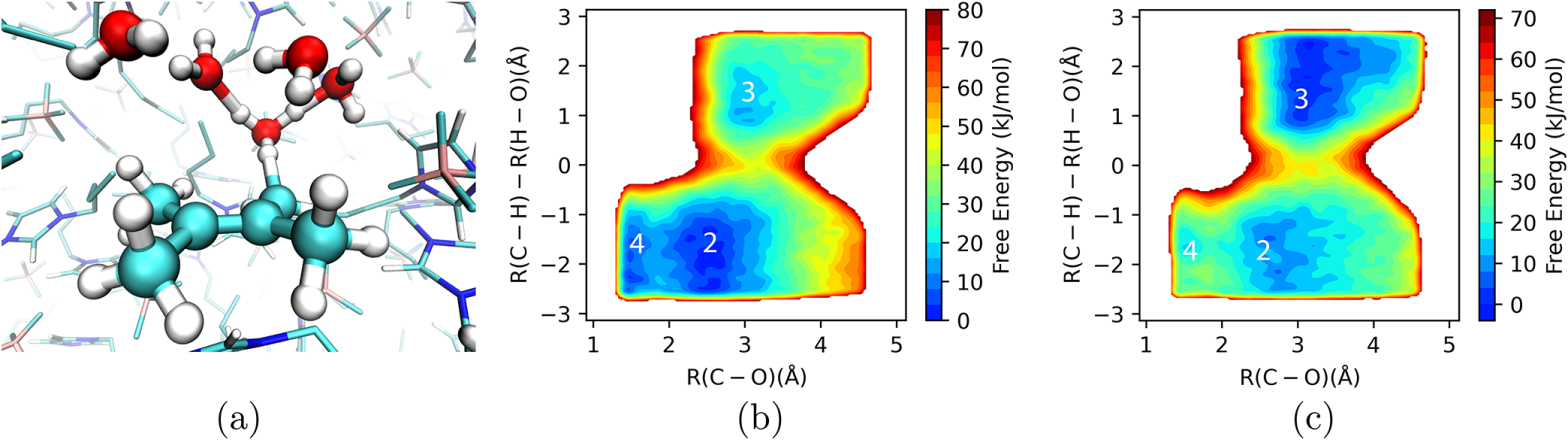
Reaction free energy profiles for tetramethylethylene cation radical encompassing nucleophilic attack and deprotonation pathway by water solvent in BMIM/BF_4_ ionic liquid; (b) for “3-water” and (c) for “5-water” microenvironments. Location of reaction intermediates **2**, **3**, and **4** is labeled on the free energy profiles. (a) Snapshot from the 5-water QM/MM simulation depicting the deprotonation transition state.

Figure 19b,c show 2D free energy profiles for tetramethylethylene cation radical reactions within the “3-water” and “5-water” microenvironments, respectively. There are clear free energy minima corresponding to the oxonium cation radical intermediate **4** resulting from nucleophilic attack, as well as allylic radical intermediate **3** from deprotonation. The free energy profiles confirm the hypothesis that the relative stability and activation barriers of nucleophilic/deprotonation reactions are modulated by the water microenvironment. While nucleophilic attack of water to **2** is facile with a low barrier within both environments, there is a substantial activation barrier for the deprotonation reaction. With increasing water content from the “3-water” to “5-water” microenvironment the barrier to deprotonation is reduced (albeit to still moderate ∼40 kJ/mol), and the deprotonated allylic radical **3** and solvated H_3_O^+^ become substantially more thermodynamically favorable. It is expected that this trend would continue with increasing water content, further reducing the kinetic barrier and thermodynamically favoring the deprotonation pathway. We should note that the deprotonation of the oxonium intermediate **4** to the alcohol (Figure 18) is observed to spontaneously occur within some of the QM/MM simulations so it is unclear whether **4** is a stable intermediate within the given water environment; this will be investigated in future work. The results suggest that within restricted water microenvironments (e.g., several water molecules), kinetic selectivity for the nucleophilic attack pathway (**2** to **4**) over the deprotonation pathway (**2** to **3**) may be achievable/possible. This could be useful, for example, for the selective electrosynthesis of 1,2-diols from olefins.^209^ We will present a more extensive computational investigation of the anodic electrolysis of olefins in ionic liquid electrolytes with dilute water in a future study.

## 5 Conclusions and Outlook

Computational methods along with software and hardware advancements have matured to enable first-principles predictions of reaction free energies and activation barriers within complex electrochemical environments modeled in atomistic detail. This perspective has focused primarily on hybrid QM/MM methodologies, which are well suited for investigating reactions at solid/liquid interfaces for which the majority of the system can be modeled at the molecular mechanics level of theory. A limitation is when the reactive substrate chemisorbs on the electrode surface, requiring quantum mechanical treatment of the solid electrode surface to capture its catalytic effect. Extensions of QM/MM that better describe adsorption at metal electrodes are under development,^173^ and in this regard, “quantum embedding” methods/algorithms may prove a promising future direction.^210,211^

The need for computational investigations of electrosynthesis reactions arises fundamentally from the extremely fast kinetics associated with the elementary reactions of electrogenerated radical and ion intermediates, with large rate constants that are very difficult or impossible to determine via standard electroanalytical characterization. These rate constants are modulated by the reaction environment (e.g., heterogeneous vs homogeneous) and reaction conditions and are important to characterize/quantify given the often numerous, kinetically competing side reactions involving the radical/ion intermediates. Within the case studies presented, we have highlighted several key mechanistic questions concerning electrosynthesis reaction pathways, for which computational investigation can play a key role in addressing. For example, residence times of electrogenerated intermediates at working electrodes are dictated by solvophobic forces, and strongly depend on nature of substrate, solvent, and electrode potential; these residence times/rate constants can be predicted directly via classical MD simulations up to an upper bound of ∼10^–6^–10^–5^ s (e.g., the length of the MD simulation) with such time scales relevant to many electrosynthesis examples given the fast nature of radical reactions. Other mechanistic questions concern how reactions of radical/ion intermediates are modulated by the electrical double-layer environment, due to its unique electrostatic and solvophobic forces and inherent steric constraints of the solid/liquid interface. In this regard, we have presented several examples highlighting strongly modulated reaction free energies and activation barriers at the electrode/electrolyte interface, as elucidated via QM/MM free energy simulations.

But all computational predictions must be experimentally verified, and this perspective has not dealt with the important question/objective of how best to experimentally benchmark/validate such computational predictions. One approach would be indirect verification based on the observed product yield/selectivity. For example, given a computational prediction that a key kinetic parameter/rate constant is modulated via a change in reaction conditions (e.g., different solvent, electrolyte), the hypothesized effect on product yield/selectivity could be tested against experimental electrolysis and product yield quantification for the different reaction conditions. Constant potential electrolysis would be preferred, with experimental measurement of the capacitance and electrode roughness factor enabling better defined experimental conditions^53^ for comparison of computer simulation predictions. The most fundamental benchmark would be a direct comparison of heterogeneous kinetic rate parameters as predicted computationally and determined experimentally. The difficulty here is the typical disparity in time scales between the large rate constants discussed in this work (e.g., *k*_D_ values in Section 3) compared to those accessible to common electroanalytical measurements.^22^ In this regard, scanning electrochemical microscopy (SECM) voltammetry in combination with rigorous data analysis/quantitative modeling has proven a powerful approach for determining thermodynamic and kinetic parameters for substrate adsorption at electrode surfaces.^212,213^ Comparison of computations to these measurements as well as first-principles voltammogram prediction,^109–111^ may be a profitable direction for benchmarking predictions of key heterogeneous rate parameters such as the desorption rate constants discussed in Section 3.
